# The (moral) language of hate

**DOI:** 10.1093/pnasnexus/pgad210

**Published:** 2023-07-11

**Authors:** Brendan Kennedy, Preni Golazizian, Jackson Trager, Mohammad Atari, Joe Hoover, Aida Mostafazadeh Davani, Morteza Dehghani

**Affiliations:** Brain and Creativity Institute, University of Southern California, Los Angeles, USA; Department of Computer Science, University of Southern California, Los Angeles, USA; Brain and Creativity Institute, University of Southern California, Los Angeles, USA; Department of Computer Science, University of Southern California, Los Angeles, USA; Brain and Creativity Institute, University of Southern California, Los Angeles, USA; Department of Psychology, University of Southern California, Los Angeles, USA; Department of Human Evolutionary Biology, Harvard University, Boston, USA; Brain and Creativity Institute, University of Southern California, Los Angeles, USA; Department of Psychology, University of Southern California, Los Angeles, USA; Brain and Creativity Institute, University of Southern California, Los Angeles, USA; Department of Computer Science, University of Southern California, Los Angeles, USA; Brain and Creativity Institute, University of Southern California, Los Angeles, USA; Department of Computer Science, University of Southern California, Los Angeles, USA; Department of Psychology, University of Southern California, Los Angeles, USA

**Keywords:** language, text analysis, hate, morality, moral foundations theory

## Abstract

Humans use language toward hateful ends, inciting violence and genocide, intimidating and denigrating others based on their identity. Despite efforts to better address the language of hate in the public sphere, the psychological processes involved in hateful language remain unclear. In this work, we hypothesize that morality and hate are concomitant in language. In a series of studies, we find evidence in support of this hypothesis using language from a diverse array of contexts, including the use of hateful language in propaganda to inspire genocide (Study 1), hateful slurs as they occur in large text corpora across a multitude of languages (Study 2), and hate speech on social-media platforms (Study 3). In post hoc analyses focusing on particular moral concerns, we found that the type of moral content invoked through hate speech varied by context, with Purity language prominent in hateful propaganda and online hate speech and Loyalty language invoked in hateful slurs across languages. Our findings provide a new psychological lens for understanding hateful language and points to further research into the intersection of morality and hate, with practical implications for mitigating hateful rhetoric online.

**Significance Statement**

Only recently have researchers begun to propose that violence and prejudice may have roots in moral intuitions. Can it be the case, we ask, that the act of verbalizing hatred involves a moral component, and that hateful and moral language are inseparable constructs? Across three studies focusing on the language of morality and hate, including historical text analysis of Nazi propaganda, implicit associations across 25 languages, and extremist right-wing communications on social media, we demonstrate that moral language, and specifically, Purity-related language (i.e. language about physical purity, avoidance of disgusting things, and resisting our carnal desires in favor of a higher, divine nature) and Loyalty related language are concomitant with hateful and exclusionary language.

*“Do not relent in purifying and cleansing the Arabian Peninsula of polytheists, heretics, and apostates.”* - Osama bin Laden (1)

Language is an indispensable tool for facilitating and establishing social connections, strengthening social institutions, and spreading ideas and culture. And yet, throughout history it has also been used to mark supremacy of the ingroup, dehumanize the outgroup, and even call for acts of hate, as exemplified in the opening quote. Indeed, language has been used to express, spread, and mobilize hatred against other social groups, resulting in intimidation, discrimination, dehumanization, hate crime, and genocide (2). The language of hate is used not only in the margins of society but is arguably foundational to certain aspects of government and religion; indeed, hateful language can be found in ancient legal documents and some religions’ texts (3). The power of language to incite hatred and spur violence is as clear today as it has been throughout history: propaganda in print and on the airwaves was used by Nazi leaders to turn a nation to genocide (4) and hateful extremists in Rwanda spurred a genocide against the minority Tutsi population via dehumanizing and incendiary rhetoric on the radio (5); and even today, rhetoric in speeches and on social media by some Buddhist monks has led to genocide against the Rohingya population in Myanmar (6). Clearly, language is too often subverted by hateful individuals and groups to harm outgroup members.

The power of language to incite hate-based violence is accompanied by the threat it poses to safe and civil discourse: online social media are infected by hate speech targeting ethnicity, gender, and other social identities (7, 8), contributing to the spread of hateful ideology with a direct negative impact on its targets (9). The problem posed by the language of hate, in all its forms, is uncontroversially unsolved. The functions of such language remain largely understudied, but researchers have pointed to a number of social functions such as prejudice perpetuation, maintenance of status hierarchies, legitimization of violence against the outgroup, norm compliance, and ingroup cohesion (10). Societies and governments often attempt to combat hate speech through structural means, including sweeping censorship by social-media platforms (e.g. Facebook’s banning of the Myanmar military (11)), “deplatforming” (12), and criminalization (e.g. (13)). Notably, most Western democracies have passed legislation against hate speech, but the United States remains an exception, with hate speech being protected under the First Amendment Right to free speech. Although, many aspects of such structural approaches are controversial; indeed, the merit of censoring hate speech, versus allowing unfettered free speech, is a constant debate among experts (14).

In the last few years, the problem posed by online hate speech has motivated a wave of research in Natural Language Processing (NLP) toward its automated detection and removal (see (15), for a review). Understandably, the vast majority of computational studies in this area have the objective of effective, unbiased detection of hate speech (or language resembling hate speech). But like ripping away a bothersome weed at the surface, structural methods lack lasting power due to their failure to target the problem at its root. Recently, some scholars have made the case that such major effort in hate-speech detection may be better directed toward building holistic solutions, which requires a deeper understanding of the antecedents of online hate, rather than methodological problem-solving; in short: detection is not a solution (16).

Despite the harms posed by the language of hate across history, cultures, and contexts of usage, there has been little scientific advancement in understanding the situational and psychological forces contributing to its use. As long as a deeper understanding of why and how individuals and collectives use language for hateful purposes eludes us, a long-term solution to the problem will as well. Based on the idea that the language of hate must be understood in order to be mitigated (10, 17–20), the present work aims to situate this usage of language in the broader context in which hate may be motivated, framed, explained, and called for: morality.

In general, moral values are strongly implicated in intergroup prejudice (21). Typically, this is a positive association, with the idea that morality—i.e. concerns about right and wrong—helps to combat prejudicial attitudes and actions. This is supported by research in psychology: works such as Rutland et al. (22) have proposed that morality plays a crucial role in children’s development of prejudice. Specifically, by way of an interaction with the emergence of group identity, children variably apply their “emerging beliefs about fairness, inclusion, and equality” (p. 279). However, the positive influence of morality on prejudice—e.g. its reduction when applied during childhood development—is potentially at odds with the wider literature on morality and its potentially deleterious consequences. Specifically, moral motives may actually be a driver in acts of violence, rather than a pacifier (23). In the same vein, humans’ moral motives may drive them to out-group hatred and, consequently, hate-based behaviors including violence and forms of hate crime (24). Recent work has suggested such a connection (25), theorizing that moralized threats are a key instigator of acts of intergroup hate. There is now an emerging literature demonstrating that acts of hate and genocide are not committed due to lack of awareness, but because the perpetrators believe that “what they are doing is right” (26).

Here, we investigate the concomitant nature of morality and outgroup hate as it occurs in language. Language affords a unique window into the morality–hate connection. Indeed, language is reflective of thought (27). Contained within instances of hateful language are the traces of hateful motivations, worldviews, and rationales leading individuals to espouse hateful beliefs. But also because while psychologists have long theorized as to the nature of hate (28), assessing hateful attitudes toward other groups of people is hampered by the shortcoming of surveys and self-reports (e.g. desirability effects; (29)). By moving away from self-reports and toward naturalistic observations of behavior, we investigate the psychology of hate and the pathways between hate and violence.

##  

### Overview of the present research

In the present work, we aim to test the hypothesis that outgroup hate and morality are concomitant in language. This broader hypothesis—that the act of verbalizing hatred on account of another person or group’s identity involves a moral component—is motivated by three different lines of thinking. First, is the idea that the development of hate is influenced by moral factors in terms of establishing group boundaries, expressing the moral superiority of the ingroup and the transgressions (e.g. betrayal, cheating) or deficiencies (e.g. impurity) of the outgroup, and the moralization of the threat of the vices that are inherent in the outgroup (26). In each of these stages of the development of hate, language, and specifically, moral rhetoric is critical to the expression and communication between members of the ingroup, and the conversion of moral concerns into outgroup hate. Second, moral rhetoric has the power to influence and persuade others toward hate. Moral rhetoric (or framing), which research has shown can impact positively (e.g. influencing attitudes about donations; (30)) and negatively (e.g. leading to violence at protests; (31)), can potentially have a role in moralizing prejudicial attitudes itself, and lead to hateful actions. Moralization can not only provide justification for a belief, but it can also make the belief more absolute and less subject to reason (32–34), while at the same time making violation of it less tolerable; it fosters a feeling that something “ought” to be done one way or the other (35). This type of rhetoric, grounded in transcendent authority, has a powerful impact in mobilizing the masses to achieve the desired ends (36). In the case of hatred, moralized prejudicial rhetoric can be used to persuade and mobilize others to commit acts of hate against the outgroup. And third, language, particularly of the moral variety, has a significant impact on the spread of messages and ideas in social networks (37–40). During intergroup conflicts, hate often spreads fast within social networks (41), and the moralized component of hateful language can theoretically influence the robust spread of it. In summary, we posit that morality is a core component of outgroup hate as it contributes to its legitimization, is used as a tactic for amplifying prejudicial attitudes through framing and rhetoric, and leads to the spread of hate in network contexts, especially when people are in morally homogeneous environments (42).

Building on prior work on the morality–hate relationship, in particular Hoover et al. (25), we approach this investigation through a pluralistic view of morality. We use Moral Foundations Theory (MFT; (43, 44)) as our theoretical framework. MFT is a pluralistic, descriptive account of morality, which posits that (at least) five foundations—Care, Fairness, Loyalty, Authority, and Purity—have contributed to solving adaptive problems throughout humans’ evolutionary past. In addition to a wide array of studies showing the predictive validity of MFT (e.g. (45)), it has also been applied in various text analytic studies (e.g. (46–49)) to study moral concerns as they manifest in various contexts (for a review, see (50)). In addition, while the morality–hate link has been observed in prior experimental studies, we ask what moral foundation in particular is most robustly concomitant with intergroup hate in language. As such, MFT is a fitting framework for the study of the moral language of hate.

In three studies, we use NLP techniques in order to quantify hate and moral foundations in language across diverse contexts. Overall, our approach to operationalizing hateful language follows both from definitions and treatments of intergroup hate in psychology, political science, legal scholarship, as well as NLP research on hate speech. In scholarship on the subject, intergroup hatred is most often studied in conjunction with intergroup violence (e.g. (51)); understanding hatred in language, though, requires understanding the myriad ways in which words can be used to accomplish the goals of intergroup hate (17). We specifically rely on three main operationalizations of outgroup hateful rhetoric: (i) language used to incite genocide (52) (ii) identity-based prejudicial language (53), and (iii) dehumanizing language (17).

Each study, in the present research, focuses on one of the above operationalizations of the language of (outgroup) hate. First, we focus on genocidal language by conducting a historical analysis of hateful language in state propaganda—specifically, speeches and texts written by leaders of the Nazi party between 1933 and 1945—which aims to quantify the influence of morality on speech that aims to persuade and incite violence. In this study, Nazi propaganda is analyzed with respect to the types of moral language evoked when discussing the outgroup (i.e. Jewish people) and the ingroup (i.e. German people). Next, we focus on identity-based hatred, and given the fact that identity-based hatred is not specific to any one language or culture, in Study 2, we present a cross-linguistic (N=25) analysis using multilingual word embeddings. Lastly, Study 3 focuses on the dehumanization aspect of outgroup hateful language by performing a large-scale analysis of the corepresentation of language of human degradation and call for violence, and moral rhetoric across social-media posts in English (N=5,937,000) on the far-right platform, “Gab.”

## Study 1: A historical analysis

Social psychology is primarily the study of contemporary history. As such, it would be myopic to maintain disciplinary detachment from historical analyses of human phenomena (54). Researchers in intergroup relations and prejudice have also recently made the case that psychologists need to incorporate historical analysis into research on racism and other social issues (55). NLP techniques provide a strong methodological toolbox to study social psychological processes using historical corpora (56). Indeed, hateful language has had a prominent role throughout history, particularly in the propaganda of known genocidal movements like Nazi Germany. Propaganda, as a public act of language, is highly intentional, designed to shape perception, often state-sponsored, and arguably dangerous (57, 58). Propaganda can influence support for violence against out-groups by dehumanizing the out-group (57), mentioning historical conflicts between in-group and out-group (59), reinforcing in-group victimization (60, 61), or promoting revenge narratives (62).

Here, we aim to supplement existing qualitative research on the rhetorical aims of Nazi propaganda with a quantitative text analysis, which specifically emphasizes its possible moral component. Qualitative research on the propaganda of Nazi Germany—which is argued to be essential in motivating those who implemented the mass murder of European Jews and other victims (63)—has described the rhetoric split along moral lines of right or good (Germans) and wrong or evil (Jews), with language that dehumanized and instilled fear of the out-group Jews, while glorifying and sanctifying the in-group of Aryan Germany^1^ (64). Similarly, we investigate moral language directed at in-group members (Germans) and out-group members (Jews). We hypothesize that there is relationship between hate and morality in language, with hateful language and moral language being concomitant. Based on prior research, we also predict that Purity, Loyalty and Authority will be associated with the Jewish out-group (25, 63–65). Given the novelty of focusing on the in-group in studies of hateful language, we make no hypotheses about the morality of in-group rhetoric other than that we expect them to mirror the pattern found in out-group rhetoric.

### Methodology

#### Data collection and preprocessing

English translations of transcripts of Nazi speeches and other propaganda, ranging in time from 1933 to 1945, were collected from the “German Propaganda Archive”^2^ (66). Speeches and documents used in the present analysis were drawn from the “Speeches and Writings from Nazi Leaders” and “Racial and Anti-Semitic Material.” After extracting individual speeches and articles from each source, texts were split into sentences using the Natural Language Toolkit (NLTK; (67)), and sentences were cleaned by removing all punctuation. Finally, sentences shorter than five words were excluded due to these sentences’ higher probability of noise. In all, 264 distinct speeches and articles were extracted from the Nazi propaganda archive, containing 39,518 sentences with an average of 21.4 words per sentence. Additionally, we analyzed “Mein Kampf,” Hitler’s manifesto and blueprint for Nazi propaganda (68), which, after preprocessing in the same way as for the Nazi speeches, comprised 10,148 sentences with an average of 28.4 words per sentence. Corpora were combined for analysis; an alternative set of analyses, which considered each of the two corpora separately, is presented in the Supplementary Materials (see Figs. S2 and S3).

In order to identify passages that contained references to the in-group and the out-group, we compiled lists of terms that were empirically observed to refer to those groups in the text. Our primary method for building these lists was to sort all words in the Nazi propaganda corpus by frequency, identifying relatively frequent words that were used to refer to either group (e.g. “German,” “Jew,” “reich,” “jewry,” etc.).^3^ In order to evaluate the interrelatedness of these terms (i.e. that each term list is referring to a consistent semantic representation), we conducted a supplemental analysis using a custom word embedding model. This analysis showed that outgroup terms and ingroup terms were tightly clustered in relation to other embeddings, suggesting that each list accurately captured group references (see Fig. S1 in the Supplementary Materials).

We then categorized sentences by whether or not they included an in-group reference (e.g. “Germany”), an out-group reference (e.g. “Jewish”), neither, or both. We discarded sentences that contained both types to reduce ambiguity. For the Nazi propaganda corpus, this resulted in 2,918 out-group sentences, and 5,189 in-group sentences, 516 sentences containing both (which were excluded), and 30,850 sentences that did not fall in either of the two categories. For the Mein Kampf analysis, this resulted in 413 out-group sentences, 750 in-group sentences, 43 sentences containing both (which were excluded), and 8,942 sentences which contained neither of the two categories.

To quantify morality in language, we relied on two tools: the Moral Foundations Dictionary (MFD; (69)) and a computational technique called Distributed Dictionary Representations (DDR; (70)). DDR is an established method which builds on word counting methods (e.g. the Linguistic Inquiry and Word Count, (71)). It uses expert-defined dictionaries (lists of related words) in order to represent a particular construct in language. Instead of word counting, however, DDR relies on distributed semantics methods—namely, “word embeddings” learned from large text corpora (e.g. “word2vec,” (72); or “GloVe,” (73))—to operationalize dictionaries, such that the cosine similarity between the average word embeddings of two constructs indicates the relatedness between the two constructs. The dictionary we use in this study, the MFD, provides a lexical resource for the study of morality in text, with 10 word-lists: two each per moral foundation (vice and virtue subcategories within each foundation). We merged the vice and virtue dictionaries to form 5 word-lists of moral terms.^4^ To measure the moral content of each sentence, we computed the DDR score of each sentence with each MFD word-list. DDR scores are predicated on pretrained word embeddings, and compute the average embedding of words in the sentence with the average embedding of words in a dictionary word-list. We use the pretrained embeddings trained on Wikipedia from Pennington et al. (73).

While DDR scores give an indication a given text’s moral loading, it is a potentially noisy estimate given its reliance on precomputed word embeddings. One way to measure the validity of DDR scores is to compare them directly to annotations of texts’ moral sentiment (74), as annotated by trained coders using the process established by Hoover et al. (75). To do so, we sampled 200 segments from Mein Kampf and 800 from the Nazi propaganda archives, and had them annotated for moral loading by trained coders. We find significant correlations between DDR loadings and human annotations across all categories (see Table S2 in the Supplementary Materials).

#### Results

To measure the effect of the particular moral domain—i.e. the difference between the moral loading for each moral foundation—across in-group and out-group contexts, moral similarities were modeled at a disaggregated level. That is, a single observation was the loading on a given moral domain (e.g. Care morality) of a given sentence. A mixed model was used, with random intercepts included for each sentence. Fixed effects were included using dummy variable encodings for the moral domain (k=5) and for whether a sentence was using an in-group, out-group, or neither context. The interaction between these two effects was also included. The dependent variable in this model was the moral similarity of the sentence with the given moral domain, *z*-scored within moral domain to account for the fact that raw similarities for each moral domain have varying means and variances.

Here we report, in detail, the findings based on the group-based analysis of the combined Nazi corpus, containing both Nazi propaganda and the text from the Mein Kampf corpus. Analyses using each corpus separately are presented in the Supplementary Materials. For the mixed effects model of the effect of moral domain and sentence type on moral similarity, the intraclass-correlation coefficient for sentence-level varying intercepts was 0.674. There was a main effect of moral domain, F(4,19,6200.0)=89.206, $\eta_{p}^{2}=0.002$, P<0.001, and of sentence type, F(2,49,059.0)=43.585, $\eta_{p}^{2}=0.0004$, P<0.001, with a significant interaction F(8,19,6200.0)=144.784, $\eta_{p}^{2}=0.006$, P<0.001 (degrees of freedom approximated using Satterthwaite’s method).

Post hoc analyses of interaction contrasts were conducted using Tukey’s post hoc test. Figure 2 contains the estimated marginal means from this model. Fairness similarity values for in-group sentences were significantly higher than Fairness similarity values for nongroup (difference of 0.184) and out-group (difference of 0.211) sentences (*P*s < 0.0001). Similarly, Authority values were higher for in-group than out-group (0.250), higher for in-group versus nongroup (0.161), and higher for nongroup versus out-group (0.150, *P*s < 0.0001) sentences, and Loyalty values were higher for in-group than out-group (0.162) and nongroup (0.168) sentences (*P*s < 0.0001). This indicates that Fairness, Authority, and Loyalty concerns are generally invoked by Nazi speakers when discussing their own group. On the other side, Purity similarity values for out-group sentences were significantly higher than Purity similarity values for nongroup (0.103) and in-group (0.113) sentences (*P*s < 0.0001); inversely, Care similarity values for out-group sentences were lower than for nongroup (0.228) and in-group (0.231) sentences (*P*s < 0.0001).

**Fig. 1.**
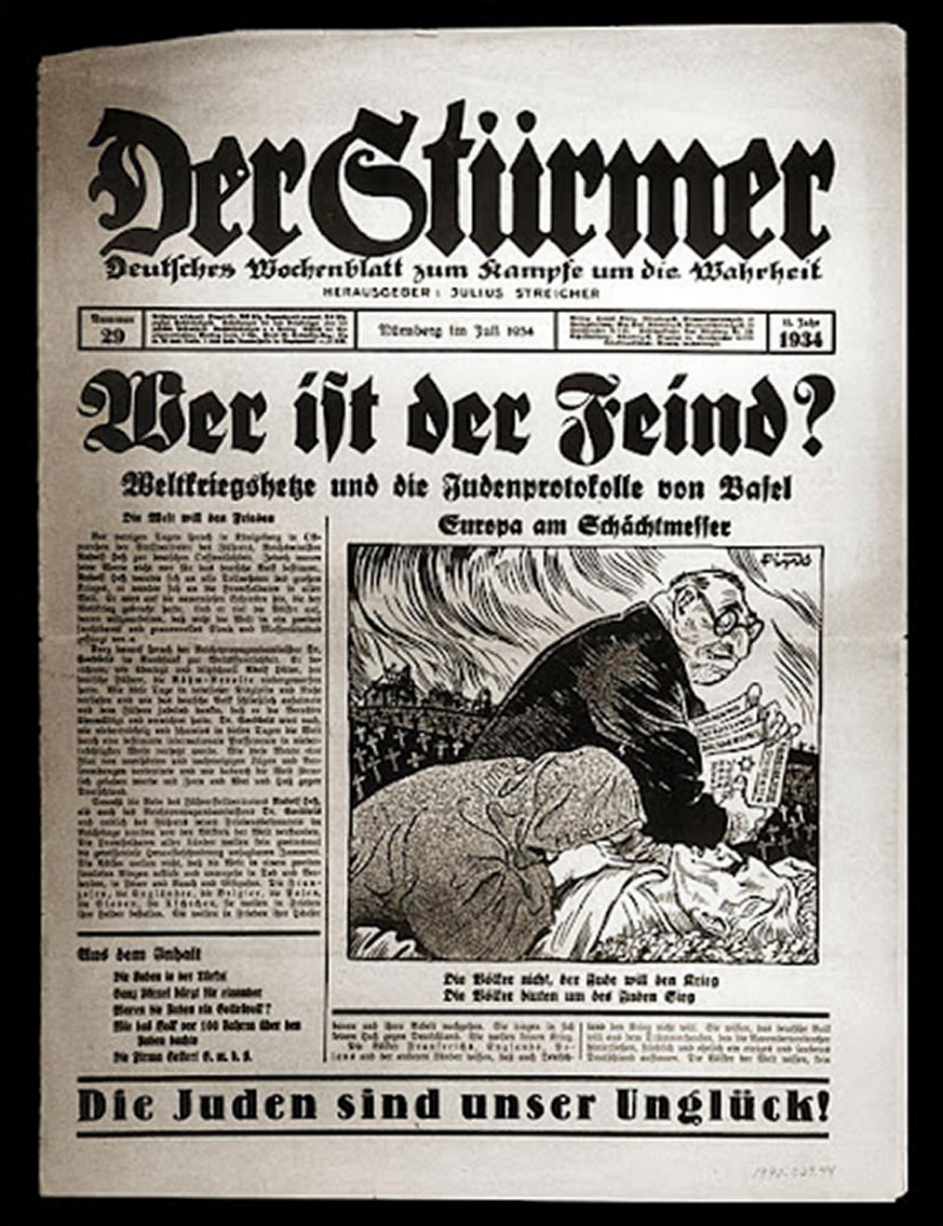
Front page of Der Stürmer, No. 29, July 1934. Der Stürmer was an antisemetic newspaper from Nazi Germany edited by Julius Streicher. Streicher was later found guilty for his influential role in inciting hatred and violence, and sentenced to death by the International Military Tribunal at Nuremberg.^5^ The top headline reads: “Who is the enemy?.” The caricature reads: “Europe under the butcher knife.” The main article blames the Jews for destroying social order and makes the claim that the Jews wanted war, while the rest of the world wanted peace. The bottom headline, “Die Juden sind unser Unglück!” (“The Jews are our misfortune”) was often used on the cover of Der Stürmer.

**Fig. 2.**
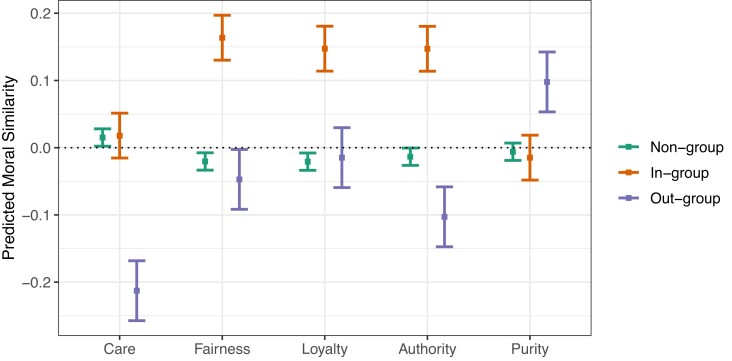
Estimated marginal means of moral similarity in the combined Mein Kamp and Nazi Propaganda corpus, for factors of moral foundations category and document category. Predicted similarities are on a standardized scale. Error bars represent estimated 99% confidence intervals after Tukey-corrections for multiple comparisons.

Having found differences in moral loading among in-group and out-group references, next we aimed to determine whether Nazi texts were moral in a general sense (i.e. when compared to meaningful corpus-level baselines). A corpus of Wikipedia sentences from articles related to the Nazi texts (e.g. articles about Germans, the Holocaust, the Jews, etc.), was used as a “neutral” reference corpus, while a corpus containing the complete King James Bible^6^ (76) was used as a “moral” reference corpus. An in-depth account of all analyses are included in the Supplemental Materials. Of particular concern is the fact that, for all moral domains, Wikipedia sentences had significantly lower moral loading than Nazi texts (P<0.0001), indicating that all content analyzed in this study is significantly more moral than average text concerning a similar topic. Nazi texts were generally less morally loaded than verses from the King James Bible, with subtle variation in this pattern (see Fig. S4 in the Supplementary Materials for visualization and details).

Lastly, in an effort to understand these effects in terms of language, we qualitatively examine the extreme ends of the moral similarity distributions for Fairness, Loyalty, Authority, and Purity. Presented in Tables 1 (in-group) and 2 (out-group) are examples, per moral category, that are at the highest end of the moral similarity spectrum for each of the two types of group-related content. All examples are in the highest three instances in the data in the corresponding category, using moral similarity scores. In order to avoid duplicates, for each sample in the data, only the foundation for which similarity was highest was considered—e.g. if Care similarity was higher than Fairness for a sentence, we considered this sentence only for the set of Care examples.

**Table 1.** Examples of moral language from in-group sentences from the Nazi Propaganda corpus.

	There would be no pity or sympathy from them because of good work or “objectivity,” no desire to do anything good for our people’s comrades.
Care	Anyone who nonetheless makes a stink, or even dares to attack or insult the Führer, must expect to lose our friendship.
	Feeling such oppression and realizing how Germany’s honor has been injured rouses their blood and awakens and strengthens their desire for liberation.
	Germany had to issue bonds as guarantees for the payment of reparations, to grant to the allies economic preferences, lost such status for herself, relinquished patent rights, etc., etc., etc.
Fairness	It is the Germany of social welfare, of social equality, of the elimination of class differences—this is what they hate!
	Where Aryan peoples rule, order and justice increase.
	These witnesses from our enemies testify to our enemies’ destructive intentions toward Germany.
Loyalty	We promise the Führer that we will remain his most obedient and loyal followers.
	Today England’s “prime minister” speaks of our Führer as traitor.
	The enemies of Aryan freedom therefore seek to replace native law with foreign law.
Authority	A racial comrade must be of German blood, without regard to religion.
	For you, doing your duty means: obey the Führer’s orders without question!
	The holy German Reich of Germanic character.
Purity	For centuries Aryan humanity protected itself against the “ferment of decomposition.” Cities and countries had strict measures for racial purity.
	Unrestrained hatred, bestial lust for destruction, a wretched desire for revenge, diabolic lust for destruction and a political depravity lacking all sense of history or instinct are mixed together in this list of measures for crippling Germany.

Examples consist of the three most highly “loaded” terms for each foundation, with loading scores computed by similarity to the respective MFD category.

**Table 2.** Examples of moral language from out-group sentences from the Nazi Propaganda corpus.

	The Jews have never done us any harm…
Care	You Jews must think your host peoples are stupid!
	It also shows why healthy peoples in every age have responded to the Jews with disgust and loathing, often enough expressing their feelings though deeds.
	To conceal its true aims, it used the slogan of “equality, freedom, and brotherhood.” Under Jewish leadership, Marxism wants to unite everything “that has a human face.”
Fairness	[“]And there must be legal guarantees against any form of discrimination against the Jews.”
	Therefore, liberalism demanded equality for all, the same opportunities for everyone, in particular the Jews, equality and freedom in the economic sphere, etc.
	Worn down, their souls crushed, they accepted Jewish doctrines that denied the fatherland and opposed all that was nationalistic.
Loyalty	The Jews promoted boycotts of party comrades.
	Formerly, anti-Jewish statements could be prosecuted only as seditious statements.
	The Jew cleverly allowed them to enjoy their sins and all who accepted these dubious values obeyed him.
Authority	The Talmud gives Jew permission to do anything he likes to gentiles, without any punishment.
	The Jew knows no morality, no decency, and he has no conscience.
	He uncovers Jewish methods of betrayal, uncovers the parasitic principle of concealment.
Purity	Jewry is the embodiment of materialism, the epitome of sensuality, of greed, of dishonesty, of selfishness, of heartlessness, and the lust for power.
	But the Jew always values and cultivates human stupidity.

Examples consist of the three most highly “loaded” terms for each foundation, with loading scores computed by similarity to the respective MFD category.

### Discussion

In this study, we found that Nazi texts were heavily moral in a general sense, and that in-group rhetoric drew specifically on Fairness, Loyalty, and Authority while out-group rhetoric drew on Purity while possessing a markedly lower amount of Care and Authority language. Based on a qualitative examination of highly moral sentences from the corpus, the influence of Purity in out-group rhetoric manifested as animalistic dehumanization and rhetoric concerning the moral and spiritual failings of the out-group. In the case of in-group rhetoric, especially moral sentences contained the positive ideals of a fair and just society (Fairness), commitments in favor of the group and against the out-group (Loyalty), and declarations of fealty and confidence in leadership and the danger posed by the out-group (Authority).

These findings suggest that the connection between morality and the language of hate exists in propagandist movements in history. In addition, by focusing on the in-group we were able to see new patterns associated with hateful movements such as the focus on loyalty and authority (see research on Nazi authoritarian personality, (77)). In contrast to our predictions, the Individualizing foundation of Fairness was associated with references to the in-group. Past research has shown that propaganda that focused on past atrocities increased justifications for the use of violence (65) which may help explain why Fairness language was associated with the in-group (i.e. the past injustices against Germany in WW1 justify violence toward the alleged guilty party, the Jewish out-group). Additionally, the qualitative results presented in Table 2 suggest that Nazi propaganda pushed a narrative of “real” justice, in comparison to the unjust transgressions committed against the in-group Germans.

## Study 2: A cross-linguistic analysis

Study 1 probed the relationship between morality and hate by conducting a quantitative study of Nazi texts. In doing so, the concomitant nature of the language of morality and the language of hate—as expressed in Nazi propaganda—was established. Here, to supplement the genocidal axis of out-group hate, we investigate the morality–hate link cross-linguistically along a identity-based axis.

We approach the cross-linguistic measurement of the moral dimensions of hateful language by considering the distributional semantics of hate across a large sample of languages. In computational linguistics research, distributional semantics refers to research proceeding from the distributional hypothesis, which is that “You shall know a word by the company it keeps” (78). In quantitative terms, the meaning of a word can be determined by deriving vector representations of words that preserve similarities between words that are used in similar contexts (see (74)). In recent years, word embeddings have become a revolutionary way to derive vector representations of words across languages (e.g. (72, 73)). Word embeddings trained on massive corpora, including Wikipedia text and text from the “Common Crawl,”^7^ reflect the semantic space of words, specifically the similarities between groups of words’ usage patterns. There has been an emerging line of work within the social sciences investigating the relation between various categories of words, through their distributional representation, to gain insights into the social context in which the words are used (e.g. (79–82)).

For this study, we use word embeddings, trained on the aforementioned corpora, for a multitude of languages in order to measure the “moral loading” of identity-based hateful terms (e.g. slurs or epithets) in those languages. Using an established lexicon of moral terms, and a lexicon of hateful terms, we measure the moral loadings of hateful terms and compare the loading onto different moral domains.

### Methods

We use the set of lexica from “Weaponized Word,”^8^ a successor to the previously maintained “HateBase” website,^9^ which contains lexica of 7,540 hateful terms from over 130 languages. The Weaponized Word uses “dynamic dictionaries of known vocabulary, threats, phishing templates and disinformation sources, as well as an understanding of negative language patterns, to provide an unparalleled lexicographic defense to content threats” (83). From the four available lexica (“Discriminatory,” “Derogatory,” “Threatening,” and “Watchwords”), we selected terms under the Discriminatory category, following qualitative inspection of words in each category and the determination that terms in the Discriminatory category were most closely related to identity-based out-group hate, versus common insults and threatening language. Terms from all available languages were accessed using the website’s API. In some cases, terms appeared in a given list that were clearly not of the specified language (e.g. an English term such as “Jew” was in the Arabic lexicon). In these cases, the words were excluded. Additionally, languages were excluded if they did not have at least 10 valid terms, resulting in only 25 of the original 130 languages for analysis. This exclusion was necessitated by the high probability that short lists of slurs (e.g. one or two terms) introduce noise, and that having multiple terms reduces this noise and allows us to find the true moral loading of slurs in a given language. Additionally, in one of our analyses we made use of labels for a given term corresponding to which social attribute the term was about (e.g. religion, ethnicity).

The main goal of the present study is to evaluate the moral “loading” of hateful terms from the Weaponized Word lexicon; to measure the moral loading of these terms, we rely again on the MFD (69). More specifically, we relied on the “seed words” for MFD introduced by Garten et al. (70) for the purpose of computing moral loadings of terms and documents using DDR (see Study 1 for a description of this method). Computing the moral loading of a given term or document using word embeddings is negatively impacted by using a large, nonspecific set of words (i.e. the entire set of words for a given dictionary such as “CareVirtue” words in the MFD). Garten et al. (70) found that a small number of seed words was sufficient to measure the aggregate similarity of a word or document to a dictionary’s embedding. We use the seed words for the five moral vices, giving us exactly 4 moral words per category. The original list of MFD words, which are in English, was then translated to the other 24 languages in our dataset of hateful terms by means of a professional translation service. These translations are provided in full in the Supplementary Materials (see Table S3).

As discussed before, to capture and represent the semantic meaning of words, we use word embeddings. Word embeddings encode the meaning of the words such that the words that are closer in the vector space are expected to be similar in meaning. Specifically, we use multilingual word embeddings trained by Grave et al. (84), which used a modified version of the FastText algorithm for training word vectors from large text corpora (85). The FastText method for training word embeddings operates on the previous continuous skipgram method of Mikolov et al. (72), and utilizes sub-word information by operating at the level of character *n*-grams. Thus, performance of FastText embeddings generalizes to languages with diverse morphology. In the present study, we extract the FastText embeddings—via the pretrained set of embeddings,^10^ which are trained on a combination of Wikipedia and Common Crawl data—of terms in the Weaponized Word lexicon, for the 25 languages which are available.

Finally, using the lexicon of hateful terms from Weaponized Word, the MFD seed words in 25 languages, and the word embeddings in each respective language generated via FastText embeddings, we computed the pairwise cosine similarities between hateful terms and moral terms.^11^ The cosine similarities of each hateful term with words from a given MFD category were averaged, yielding a single (average) similarity score per MFD category, language, and hateful term. Using word embeddings’ cosine similarity between two different categories of words in order to determine the “loading” of one onto the other is a practice established in prior research (70, 80–82, 86).

### Results

For moral loadings of hateful terms across 25 languages, a mixed effects model was estimated which measured the fixed effect of the MFD category on the similarity between a given MFD word and a given hate word. Random intercepts of language were included, and similarly random intercepts for each moral word, across translations of the word into other languages, were allowed to vary.

We used this model to answer two questions. First, we sought to measure the relative differences in moral loading of hateful terms among the moral foundations. Second, as a robustness check, we sought to understand how this relation is differed from the loading of hate across common categories of language.

To perform these analyses, we relied on post hoc pairwise comparisons of moral foundations. To examine the relative differences among moral foundations, pairwise comparisons of estimated marginal means were used. This revealed significant pairwise differences, detected using a Tukey correction for multiple comparisons. Importantly, we find that hate words’ similarity to Purity was significantly higher than words related to all other foundations (*P*s < 0.001). Additionally, hate words’ loading onto Care was lower than for all other foundations (*P*s < 0.001). No other significant differences were observed.

**Fig. 3.**
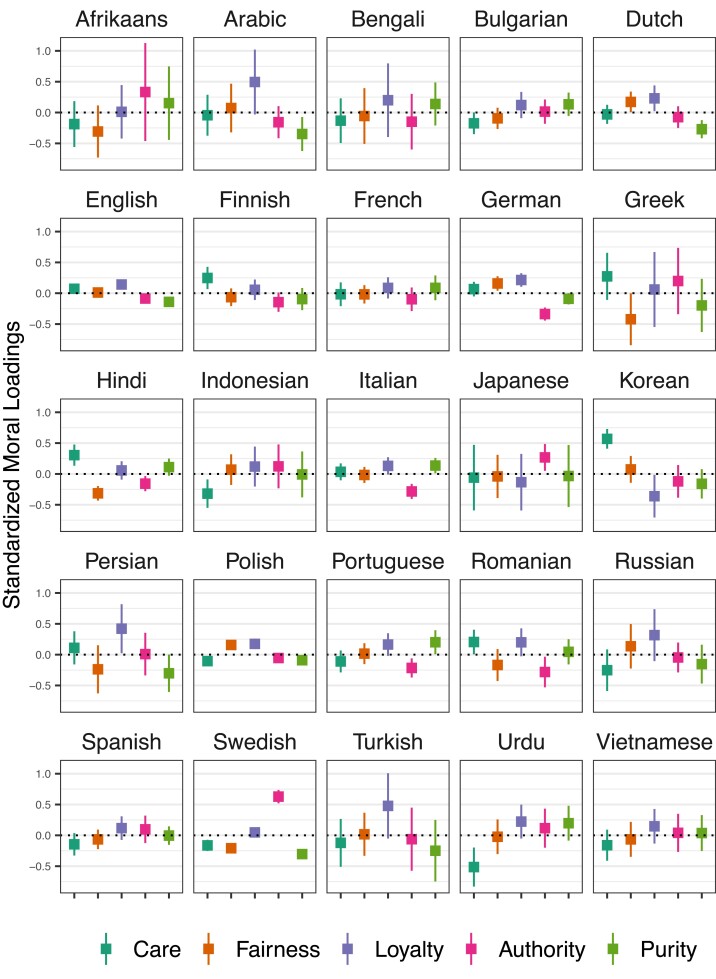
Average moral loadings (with confidence bars twice the standard error) of hateful terms from Weaponized Word, per language. Moral loadings were first standardized within language.

In Fig. 3, the distribution of moral similarities, by foundation, are shown across the 25 languages. All reported statistics have been standardized within language for interpretability. From this disaggregated view, it is apparent that, although overall hateful terms load onto Loyalty, there is substantial heterogeneity among languages. For example, for certain languages (e.g. Arabic, English, German, Persian, Russian) hateful terms load highly onto Loyalty, while the opposite is true for other languages (e.g. Hindi, Japanese, Korean). Certain languages exhibit other patterns; for example, hateful terms in Finnish, Hindi, and Korean are highly loaded onto Fairness, while hateful terms in Japanese and Swedish are highly loaded onto Authority. Notably, the confidence level and size of differences among foundations varies by language, which has to do with the number of hateful terms available for a given language, as this can increase the noise in the sample.

We also include an analysis of the targeted groups of each hateful term included in this dataset. Nearly all terms (∼99.1%) in the dataset of hateful terms is annotated with the group of people which are targeted by a given term (e.g. religious, ethnic, nationality). For our analysis, we excluded terms with multiple target annotations (∼18.5%) and those with no target annotations (∼0.9%). As the presence of these target group annotations is unevenly distributed across languages—many languages had exclusively one target group while certain languages, such as English, Finnish, and German, had many groups represented—here we did not explicitly model the language of terms as an independent variable. To model the relationship that the target group and moral foundation had on moral similarity of hateful terms, the group-standardized similarity scores were modeled as the dependent variable, while the target group (“class,” “disability,” “ethnicity,” “gender,” “nationality,” “religion,” or “sexual orientation”), the moral foundation, and their interaction were included as independent variables in a linear regression.

**Fig. 4.**
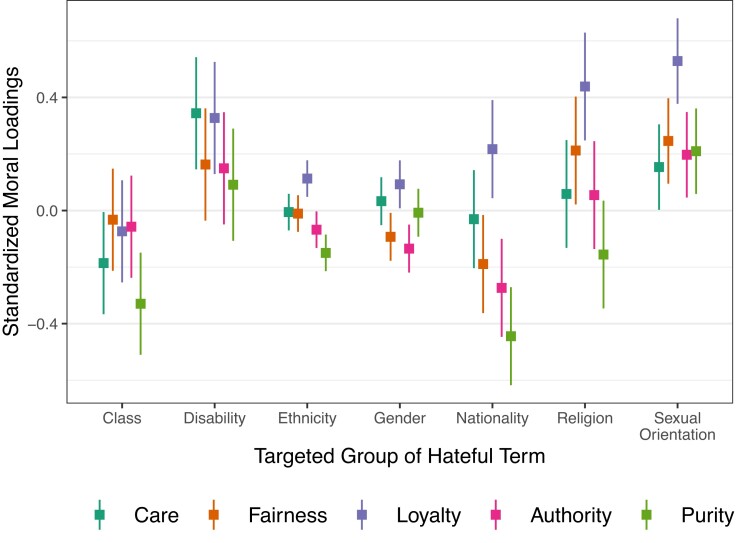
Estimated marginal means for the model predicting moral similarity of hateful terms from the targeted group of the given hateful term. Error bars represent 95% confidence intervals after Tukey-correcting for multiple comparisons.

There were significant main effects of target group, F(6,10,270)=19.723, $\eta_{p}^{2}=0.011$, P<0.001, and foundation, F(4,10,270)=21.381, $\eta_{p}^{2}=0.008$, P<0.001, with a significant interaction, F(24,10,270)=1.963, $\eta_{p}^{2}=0.005$, P=0.003. To measure the relative effect of moral foundations and targeted groups on moral similarity, we used Tukey-corrected post hoc contrasts and estimated marginal means, the latter of which are shown in Fig. 4. No significant differences in moral loading were observed between foundations for the “Class” or “Disability” target groups. Within the “Ethnicity” target group, Care (P=0.017), Fairness (P=0.024), and Loyalty (P<0.0001) had a higher loading than Purity, while Loyalty also had a higher loading than Authority (P<0.0001). Within the “Gender” target group, Care had a slightly higher loading than Authority (P=0.048) while Loyalty had a higher loading than Fairness (P=0.020) and Authority (P=0.002). For “Nationality,” Loyalty had a higher loading than Fairness (P=0.010), Authority (P=0.001), and Purity (P<0.0001), with Care higher than Purity (P=0.008). For “Religion,” Loyalty was slightly higher than Care (P=0.045) and Authority (P=0.042), while Loyalty was higher than Purity (P<0.0001). Finally, for the “Sexual Orientation” target group, Loyalty had a higher moral loading than Care (P=0.005), Authority (P=0.020), and Purity (P<0.029).

As a robustness check, and to examine the moral profile of hateful terms in relation to other categories of language, we translated terms from all the “content-word” categories (N=48) of the Linguistic Inquiry and Word Count (LIWC; (71)) in the same languages as in our analysis, and computed hate loadings of each category by randomly down-sampling terms from each category in LIWC. Similarities were computed between the LIWC categories and hate as before. This analysis revealed, most prominently, that except for five categories of *Female references*, *Comparison*, *Anger*, *Biological processes*, and *Home*, hateful terms have a significantly higher Loyalty loading than rest of the LIWC categories across languages (see Fig. S5 in the Supplementary Materials for details).

### Discussion

At multiple levels of analysis and across a diverse set of languages, hateful terms load predominately onto the Loyalty dimension. Probing the hate–morality relationship with respect to which social group was targeted by the hateful term, we found that the high Loyalty loading of hateful terms primarily has to do with ethnicity, nationality, religion, and sexual orientation. The cross-linguistic finding that hateful terms load heavily onto Loyalty language contrasts with the finding of Study 1, which found that Purity was by far the strongest predictor of the presence of hate-based rhetoric in Nazi propaganda wherein one particular ethnic identity (i.e. Jewish people) was markedly derogated by political figures.

## Study 3: Moral dimensions of online hate-based rhetoric

One way in which hatred is currently wielded in language is in online hate speech. Though humans have likely engaged in hateful rhetoric throughout history, hate speech has more recently become explicitly recognized as a legal and societal issue, with considerable amount of resources being dedicated, both in the private and public sectors, for controlling its spread and mitigating its real-world effects (e.g. domestic terrorism). In this study, we present an analysis that aims to measure the degree to which hate speech is dependent on the moral language expressed in the text using data from the alt-right social-media site “gab.com.” Gab is celebrated for its endorsement of free speech and has attracted a multitude of self-identifying “far-right” users (87, 88). Unlike most mainstream social networking sites, Gab permits its users to post nearly anything, including hate speech, making it a fitting setting in which to study the dynamics of hate speech in a real-world setting.

Using expert-assigned hate speech labels generated in prior work (17) and additional moral foundations labels generated for this study, we train machine learning models to predict hate speech and moral foundations labels. Each of these models was then used to predict the presence of hate speech and each moral concern in a large Gab corpus as used by Cinelli et al. (89) and compiled by Gaffney (90), $N_{\mathrm{posts}}=13,020,612$, $N_{\mathrm{users}}=65,375$, after removing small posts (i.e. those with too few English tokens) and Gab users with too few or too many posts. Finally, we assess the dependency between hate speech and moral labels using hierarchical logistic regression with random effects for Gab users.

### Methods

#### Operationalizing and annotating hate speech and moral rhetoric

Here, we build on prior research (17), which both operationalized hate speech as specifically *hate-based rhetoric* and used this operationalization to create the “Gab Hate Corpus” (GHC), a large corpus (N=27,655) of manually annotated posts from gab.com. Distinguishing hate-based rhetoric from popular conceptualizations of hate speech helps to address the ambiguity surrounding the term “hate speech” across legal, scientific, and practical settings (91). Free speech laws in the United States protect substantially more acts and classes of language than in countries such as Canada, the Netherlands, and Germany, in which culturally specific rhetoric (e.g. Holocaust denial) are outlawed and language is evaluated by its intention to harm or incite further violence or hatred (14). To address these complexities, the annotation guide relies on these latter countries’ definitions of hate speech to operationalize “hate-based rhetoric,” a construct that captures language that derogates, dehumanizes, or incites violence against a protected social group (see (17) for detailed discussion of this construct). Hate-based rhetoric encapsulates two types of rhetoric: Human degradation (HD) and Calls for Violence (CV), demarcated by their respective intents and the former’s focus on the general attack on the dignity of specific groups of people.

Using the Moral Foundations Coding Guide and text annotation methodology developed by Hoover et al. (75), we also annotated 27,655 posts for moral foundations content. For each of the coding tasks, annotators were first trained using a coding guide, passed an annotation-based test of their understanding of the different categories, and performed annotations on Gab posts using a custom user interface. Each of the posts in the GHC were labeled for moral foundations, in addition to hate-based rhetoric, by a minimum of three annotators (e.g., see Fig. 1). To illustrate the type of language which is jointly hateful and moral, we have included a set of examples for each category in Table S4 in the Supplementary Materials.

To evaluate annotator agreement, for the hate speech and moral vice labels, we calculated Prevalence-Adjusted and Bias-Adjusted Kappas (PABAK; (92)), which adjust for prevalence and bias in the rate of positive examples (75). All PABAK Kappas were at least at the level of “substantial agreement” (0.6–0.8 (93)): 0.75 (Care), 0.81 (Fairness), 0.79 (Loyalty), 0.82 (Authority), and 0.83 (Purity). Intercoder agreements for hate-base rhetoric of this corpus are presented in Kennedy et al. (17).

#### Labeling of a large Gab corpus via machine learning

The GHC annotations and the moral foundations annotations were used to train machine learning classifiers. Annotations were aggregated into binary labels at the post level by majority vote, with ties (for posts with an even number of assigned annotators) settled by assigning “positive” to a given label. After aggregation, a variety of NLP methods were applied in the classification of each binary label (HD, CV, Care, Fairness, Loyalty, Authority, and Purity). Most prominently, a state-of-the-art method for predictive modeling on text was applied in order to generate predicted labels for the entire set of Gab posts: “fine-tuning” Transformer-based language models, which are previously fit to massive corpora of text, on our particular task, which in this case is text classification. Specifically, the Bidirectional Encoder Representations from Transformers method (BERT; (94)), having been pretrained on text from Wikipedia and from a large corpus of books, was fine-tuned (in turn) to each classification task by the procedure outlined by Devlin et al. (94) and implemented using the transformers (v2.6.0) library in the Python programming language (v3.6).

In addition to fine-tuning BERT, two baselines were also implemented, as robustness checks, both of which featured “Support Vector Machines” (SVM; (95)), a machine learning technique often used when model inputs are count data from text corpora. SVM models were first paired with features extracted from Gab posts using the dictionaries from the Linguistic Inquiry and Word Count (LIWC; (71)). These 73 word categories cover a wide range of word types in English, including grammatical types as well as different topics (e.g. family, time, emotions). The presence of words from each LIWC category was counted in each document and normalized by the document length, and the feature set was used as input to a binary SVM classifier implemented in Scikit-Learn (96), using linear kernels and optimizing for the “C” parameter (controlling amount of regularization) and accounting for the imbalance of class labels using the “class_weight” parameter. In an identical way, Gab posts were converted to numerical features using Term Frequency-Inverse Document Frequency (TF-IDF (97)), an established baseline for text representation used in NLP which normalized a given word count by its relative infrequency across the document corpus.

Each of the three approaches was evaluated using cross-validation, and the full description of results and model fitting are given in Table S5 in the Supplementary Materials. In general, we found that the fine-tuning provided the best performance in terms of $F_{1}$ score, which balances model precision and recall. However, the results of the other methods are also provided as robustness checks.

##### Analytic procedure

For each of the HD and CV hate-based rhetoric labels, one model estimated the probability that a given post contains HD or CV (respectively) as dependent on whether or not it contains each of the five moral vice labels, treated as distinct variables due to their being mutually nonexclusive. To account for the fact that this corpus contains multiple messages per user, each model was estimated using a mixed effects logistic regression with varying intercepts and slopes (for moral vice labels) for each user.

We aimed to include regular users of the Gab platform (i.e. excluding users that rarely posted) and exclude accounts that were likely bots based on their posting behavior (e.g. news aggregator accounts with thousands of automated posts). Posts from users without at least 10 posts were excluded, comprising 380,716 posts from 145,183 users (μ=2.62). Furthermore, users with more than 500 posts were excluded (18,464,516 posts from 6,714 users, μ=2,750.15), leaving 4,234,535 posts from 59,544 users. As a robustness check, an additional set of models was fit with a more lenient threshold, with users excluded if they had at least 5,000 posts (removed 10,059,155 posts from 883 users, μ=11,392.02), leaving 13,020,612 posts from 65,375 users. This additional set of models is described in the Supplementary Materials and reported in Table S6 and did not contain notable differences from the main set of models presented here.

#### Results

The results from our analysis are shown in Table 3. Two models are shown, with DVs of the binary presence of Human Degradation (HD) and Calls for Violence (CV), as predicted on the large corpus of Gab posts using fine-tuned BERT models. The use of varying intercepts for Gab users was justified by high intraclass correlation coefficients (ICCs), with ${\mathrm{ICC}}_{\mathrm{HD}}=0.365$ and ${\mathrm{ICC}}_{\mathrm{CV}}=0.288$. Each model was fit with 5 fixed effects, one per moral vice category (as predicted by fine-tuned BERT models), and slopes for each moral vice category were allowed to vary at the user-level.

**Table 3.** Results from two hierarchical logistic regressions of Human Degradation (HD) and Calls for Violence (CV) labels, with fixed and varying effects for each of the five MFT (vice) labels. All fixed effects were significant (P<0.001).

	Dependent variable:	
	Human degradation	Call for violence
Fixed effects		
Intercept	− 3.359 (0.006)	− 7.026 (0.012)
Care (Harm)	1.187 (0.005)	3.982 (0.012)
Fairness (Cheating)	0.884 (0.005)	− 0.643 (0.020)
Loyalty (Betrayal)	1.321 (0.013)	1.402 (0.027)
Authority (Subversion)	0.142 (0.013)	− 0.490 (0.039)
Purity (Degradation)	2.649 (0.005)	0.328 (0.012)
Random effects		
Intercept${}_{\mathrm{User}}$	1.584	1.060
Harm${}_{\mathrm{User}}$	0.162	0.417
Cheating${}_{\mathrm{User}}$	0.150	0.179
Betrayal${}_{\mathrm{User}}$	0.450	0.790
Subversion${}_{\mathrm{User}}$	0.321	0.595
Degradation${}_{\mathrm{User}}$	0.263	0.168

*Note:* Estimates reported on the log-scale.

These results from the two hierarchical logistic regressions—one each to the predicted values of HD and CV labels—showed overall that the language of hate is related to the language of moral vices. In particular, after adjusting for the presence of other categories of morality, the presence of Purity indicated a 14-times increase in the odds of HD being present, b=2.649, SE = 0.005, Z=513.30, odds ratio ( OR) =14.14. Other categories of moral vice language were also positive, though with smaller magnitude; for example, the effect of Harm language on the presence of HD was positive, b=1.187, SE = 0.005, Z=254.39, OR=3.278. In contrast, the model of CV labels showed a different pattern, which befits the differing semantics of attacks on human dignity and voicing a desire or intent for violence. In particular, CV was approximately 53-times more likely when Care language was present, b=3.982, SE = 0.012, Z=326.36, OR=53.36. A negative effect was observed for Cheating and Subversion labels, with a small effect of Degradation, b=0.328, SE = 0.012, Z=27.47, OR=1.388, and a moderate effect for Betrayal, b=1.402, SE = 0.027, Z=52.16, OR=4.065.

Lastly, we sought to determine whether these findings, which were derived from predicted labels, differ substantially from a model fit to the annotated dataset. If the machine learning classifiers introduced a large amount of noise, then we might see differences in the main findings reported from this model. Using the annotated dataset (n=27,655), we fit similar regression models using hate labels as dependent variables and moral vice labels as independent variables. Whereas our main set of regression models included varying intercepts and slopes for Gab users, in the annotated corpus posts were randomly sampled and thus there were insignificant repeated samples per user. As such, we fit two logistic regression models, one for each hate label (HD and CV). We found nearly identical effects in the model fit to the annotated dataset as we found with the full Gab dataset with predicted labels. In particular, the strongest predictor of HD in the annotated set was Purity (β=2.684, SE = 0.067, OR=14.637, P<0.001) and the strongest predictor of CV was Care (β=4.255, SE = 0.212, OR=70.464, P<0.001). The full results are reported in the Supplementary Materials (see Table S7).

### Discussion

The evident relationship between moral language and online hate speech supports the broader hypothesis that moral language and hateful language are concomitant. In particular, when Purity language is present in a post, that post is 14 times more likely to be predicted as language that attacked human dignity. The finding that hateful language that called for violence is strongly related to Care violations, in contrast, is likely due to the fact that calls for violence often include an implicit endorsement of harm against others. Overall, these results suggest that perceptions of Purity violations can be used to motivate, legitimize, or justify hate-based rhetoric, specifically derogation of out-group members, in unregulated social-media platforms.

## General discussion

In this work, we tested the hypothesis that morality and intergroup hate are concomitant in language. We find strong support for this hypothesis across different operationalization of intergroup hateful rhetoric, with Study 1 focusing on language used to incite genocide; Study 2 investigating identity-based prejudicial language through the study of hateful slurs across languages; and Study 3 focusing on hate manifested as dehumanizing language on social media. Furthermore, our analyses contrasted the relationships between hateful language and each moral foundation, suggesting that Purity and ingroup Loyalty language are most strongly tied to hateful language.

In Study 1, we conducted, to our knowledge, the first quantitative text analysis of Nazi texts, focusing on their moral loading, computed using distributed semantic representations of an established dictionary of moral terms. Nazi texts were found to be heavily moral when compared with a neutral, related corpus (i.e. text from Wikipedia). In addition, we found that Purity language was especially prevalent in language containing references to the out-group (i.e. Jewish people) while Fairness, Authority, and Loyalty language was especially prevalent in language that referenced the Nazi in-group. In Study 2, we examined the moral loading of hateful slurs and other terms across 25 languages using multilingual word embeddings. Overall, hateful terms tended to be more aligned with the Loyalty dimension of distributional semantic space. Further, we see this relation pronounced when the hate terms target ethnicity, nationality, religion, and sexual orientation. We also examined the moral loading of hateful terms in relation to other, more general, semantic categories, captured via “content-word” categories of LIWC. This analysis revealed that in comparison to nonmoral categories, the relation between hate and Loyalty is strong across most of the 25 languages examined. Lastly, in Study 3, we tested the relationship between morality and hate-based rhetoric in the language of far-right social media, leveraging expert annotation of texts and state-of-the-art machine learning. In a corpus containing millions of posts from nearly 60,000 distinct Gab users, we found that posts containing Purity sentiment were more than 14-times more likely to contain “Human Degradation” language than those that did not contain Purity, one of the two main types of hate-based language defined by Kennedy et al. (17).

The findings from these three studies establish a clear empirical link between morality and hate in language. However, they do not shed light on the underlying psychological mechanisms at play. Further investigation into why this relationship exists is left for future work. Here, we outline possible interpretations that can structure future theoretical integration. One interpretation would be that, if hatred and morality are in fact concomitant constructs (23, 25)—i.e. that prejudicial attitudes and moral concerns draw on the same set of psychological phenomena—then their evident relationship in language is simply this concomitance manifested in verbal behaviors. Our results, and in particular results from Study 2, support this interpretation, given its broad coverage of hateful terms across languages and its use of distributed semantics to understand the widespread usage of words in a given language community. This provides empirical support for the long-standing theories in social psychology (e.g. (28)) and anthropology (e.g. (98)) that morality is the catalyzer for intergroup violence, and that hate, enacted behaviorally or through language, is inseparable from morality. Hence, morality and intergroup hate are concomitant psychological constructs, and our findings provides supports for this at the linguistic level.

Another interpretation of our findings might view morality as a mechanism for those wishing to effectively communicate, and ultimately enact, intergroup violence. In this interpretation, call for violence is framed using morality in order to gain legitimacy, spread through social networks, and to call for action. Indeed, the findings from Study 1 would imply this to be the case: Nazi propaganda had an explicit, unambiguous agenda toward hatred and violence, and any rhetorical devices found in such propaganda could be seen as tools used by the speakers to increase the magnitude of the response, and reach of the hatred in society (37). Essentially, in this interpretation, violence against the out-group is called for, and legitimized, through moral framing. In other words, those in power, frame their prejudicial motives using morality, and by doing so, instill hatred in the in-group.

The three studies in this article do not point to either of these interpretations exclusively but give supporting evidence to both. By analyzing language in multiple communicative contexts, we can perhaps eliminate the possibility that the correspondence between hateful language and moral language can be solely understood either in terms of framing or in terms of concomitance made manifest. Indeed, findings from Study 3 provide support for this interpretation. Individuals posting prejudicial language on social media have obvious communicative goals, and thus might imbue their language with moral rhetoric and framing for higher engagement and reach (40); however, the expression of hatred might implicitly draw on the moral concerns of the speaker beyond the post’s immediate wording. In conclusion, morality is used in the communication of hatred, but it is also related to hatred at a more fundamental level (23, 25).

The findings of this article extend beyond the mere strength of the association between moral and hateful language. In our analyses, we find that the language of Purity is commonly associated with hateful language. In essence, the sacred violence that hateful language calls for, as the bin Laden quote at the beginning of this article indicates, entails the purification and cleansing of the in-group from the moral disdain of the out-group. This finding further confines the hate-morality connection; hatred is often articulated through the language of physical and spiritual degradation and pollution. This type of language not only can be used to dehumanize the out-group while framing the in-group as sacred and pure (99), but it can enforce segregation and further distancing between the groups (100), which often is one of the desired ends of hate. In addition to Purity, we find evidence that Loyalty is a key moral element of hateful language, particularly as it relates to ingroup language in Nazi texts and the semantic representation of hateful terms across multiple languages. This relationship is most likely explained by role of ingroup loyalty in creating and fomenting hate (Study 1) and in the way hateful terms attack the humanity of members of the outgroup (Study 2).

Our findings have some implications for the emerging literature at the intersection of moral psychology and intergroup relations. Recently, Spring et al. (101) highlighted the utility of moral motivations for collective action. The traditional consensus in moral psychology has been that people should maintain institutions that deter harmful actions but minimize outrage since moral outrage is linked to negative outcomes. More recent work, however, has uncovered the negative side of moral motivations, even outrage. For example, experimental work using both naturally occurring outrage and induced outrage has shown that moral outrage is linked to greater support for nonviolent peacemaking policies (102). Complementing current work in merging the intergroup relations and moral psychology literatures, and consistent with the idea that “morality binds and blinds” (103, p.311), our findings allude to the double-edged sword of moral motivations: they can sometimes lead to constructive collective action (101), but they can also be used to motivate or justify some of the most heinous acts in human history such as the Holocaust, the Rwandan genocide, slavery, and terrorism, as our opening quote exemplifies.

Our findings also have practical implications for moderation of social-media platforms. While a wave of new research, both in academia and in industry, has focused on automatic detection (and removal) of hate speech (15), this task is very much unresolved. Our results indicate that hateful language is coexpressed with moral, specifically with Purity-related, language. Our work also provides preliminary evidence that negative emotions, especially anger, highly co-occur with hate-related terms. In Study 2, anger showed consistent relationships with hateful terms across languages. Given the evident links that both moral concerns and negative emotions have with hateful language, future computational research can incorporate moral and emotional signals into hate speech classifiers. Indeed, anger can be considered an essential component of “moral outrage” (104) and “moral upset” (i.e. perceived moral transgression regardless of which moral foundation has been violated; (105)). Future research is encouraged to not only investigate the role of moral upset and outrage in hate speech but also in less toxic forms of derogatory language such as “fear speech” in which people, usually from majority groups, incite fear about particular social identities (e.g. immigrants) (106).

It is well-documented that acts of hate are difficult to study experimentally, due to the inability of researchers to expose individuals to acts of hate, or ask them to participate in them (29). In this article, we relied on naturalistic data to investigate the overlap between hate and morality “in the wild.” However, our work is purely observational in nature, and the conclusions made from this work should not infer causality.

Furthermore, we point out several methodological limitations in our methodology which ought to constrain interpretations while also encouraging additional investigation. In Studies 1 and 2, word embeddings and dictionaries were paired in order to measure the moral loading of terms and sentences. While word embeddings improve the validity of using dictionaries, particularly in noisier text, the gold standard in text analysis is constantly evolving, and new techniques in NLP are emerging that might improve the validity and level of detail of our studies. We leave this to future work, which will likely reveal a more nuanced, accurate set of findings. Additionally in Study 2, we conducted a broad analysis across more than a dozen languages. This decision allowed us to discover broad, multilingual trends in the relationship between morality and hateful terms; however, text analysis that does not involve analysis by native speakers is susceptible to error due to the nuances of language. We hope that our findings encourage dedicated analysis in some of the languages included in our study. Lastly, Study 3 is a useful insight into the relationship between moral language and hate-based rhetoric, but it is limited in that it is only one particular social-media network, involving one online community with particular outgroup targets, and in one language (English). Our findings ought to encourage further investigation using more diverse corpora and studying more communities, both online and offline.

We also acknowledge that the concept of moral purity, even though predictive of various real-world behaviors (e.g. (45, 86)), is complex and may be multifaceted (107). With that said, our work is a clear illustration of the value of a pluralist-descriptive approach to human morality—one that is not confined to normatively endorsed principles in the West (108). In conclusion, we argue that investigating moral purity is central to our better understanding of the powerful, and under-investigated, destructive forces of morality.

## Supplementary Material

pgad210_Supplementary_Data

## Data Availability

The data for Studies 1 & 2 are publicly available (Study 1: https://research.calvin.edu/german-propaganda-archive; Study 2: https://weaponizedword.org & https://fasttext.cc/docs/en/crawl-vectors.html). For Study 3, the GHC is publicly available and can be accessed at https://osf.io/edua3. The moral labels for GHC have been made available and can be accessed at https://osf.io/a4rs6/.

## Footnotes

1
https://encyclopedia.ushmm.org/content/en/article/julius-streicher

2
https://research.calvin.edu/german-propaganda-archive/

3 See Table S1 in the Supplementary Materials for a full list.

4 This decision is justified by the fact that the words for a given vice category (e.g. “disloyalty” for the Loyalty foundation) are highly similar, in terms of distributed representation, to words for the respective virtue category (e.g. “loyalty”).

5 Full text accessed via Project Gutenberg, https://www.gutenberg.org/cache/epub/10/pg10.txt

6
https://commoncrawl.org/

7
https://weaponizedword.org/

8
http://www.hatebase.org/

9
https://fasttext.cc/docs/en/crawl-vectors.html

10 This method is slightly different from the DDR approach used in Study 1 but relies on the same principles and underlying logic. Here, we opted for a disaggregated approach due to an uneven number of words being available for each category across the languages examined. Pairwise similarities were computed, and inferences were made from models containing all word-level similarities rather than using category-level similarities. By doing so, we accounted for the fact that each language contained a highly variable number of words, and we reduced the probability of noise in the dictionary words affecting our inferences.

11 We will make the moral annotations publicly available upon acceptance of this article.
