# Supplementary material for: The (moral) language of hate
Source: PNAS Nexus. 2023 Jul 11;2(7):pgad210. doi: 10.1093/pnasnexus/pgad210 (PMC10335335; doi:10.1093/pnasnexus/pgad210)
Supplement: pgad210_Supplementary_Data [file pgad210_supplementary_data.pdf]

Appendix A

Study 1: Historical Analysis

Methods

In the construction of the in-group and out-group sub-corpora, lists of terms which had to due with both groups were used to divide the corpus. Single-word terms (e.g., "germany") were identified manually by inspecting a list of the most frequently used terms in the corpus and selecting those that were associated with Germans or Nazis. Multi-word terms (e.g., "german reich," "jewish question") were included due to the terms "german" and "jewish" occurring in multiple contexts. In order to ensure that these two terms were referencing the respective in-group or out-group, the 30 most frequent two-word phrases were extracted per term, and two-word terms which referenced the respective group were selected. For example, the phrase "german reich" was included, whereas the phrase "german in" was not. In addition to reducing instances of the terms "german" and "jewish" which were potentially not referencing the respective group, the list of terms provided in Table S1 gives insight into the ways in which these terms are used in the corpus.

To understand the relatedness of these terms to each other — in other words, to determine how well these terms represent a tight cluster to terms related to the same thing — we sought to compare the embeddings of these phrases to the words in the rest of the corpus. To do this, we combined the Nazi propaganda corpus and the Mein Kampf text into a single set of documents and fit a word embedding model. The word embedding model we chose was the "FastText" algorithm (Bojanowski et al., 2017). We chose FastText due to its speed and efficiency in training due to using sub-word information. A FastText model with dimension 32 was fit using the "gensim" python library (Řehřek, Sojka, et al., 2011) for 100 epochs, with a window size of 5 words and minimum word frequency of 2. Embeddings of single-word terms in each list were extracted from the model directly, while embeddings of two-word phrases were generated by adding together (element-wise) embeddings from each of the component words.

Table S1
Terms used for identifying ingroup and outgroup sentences.

	Terms/Phrases
Ingroup terms	"german people," "german state," "germany," "german reich," "german worker," "german soldier," "german nation," "german women," "german armies," "german blood," "german youth," "german military," "german troops," "german woman," "german men," "german citizen," "german christian," "german history," "german labor," "german victory," "german war," "german will," "german culture," "german economy," "german life," "german population," "national socialism," "our people," "führer," "aryan"
Outgroup terms	"jew," "jewish question," "jewish world," "jewish spirit," "jewish people," "jewish race," "jewish press," "jewish blood," "jewish rule," "jewish lackeys," "jewish bolshevism," "jewish problem," "jewish thinking," "jewish leadership," "jewish influence," "jewish nature," "jewish domination," "jewish doctors," "jewish hatred," "jewish population," "jewish hands," "jewish munitions," "jewish war," "jewish peace," "jewish chronicle," "jewish religious," "jewish newspaper," "jewish dominance," "jewish state," "jewish rulers," "jewry," "judah," "bolshevik," "zionism," "zionist"

To analyze the embeddings of ingroup terms, outgroup terms, and non-group terms, we visualized them using the UMAP dimensionality reduction algorithm (McInnes et al., 2018). The result, after down-sampling the non-group terms randomly ($k = 1000$) due to the vocabulary being to large to visualize, is shown in Figure S1. As observed in the figure, the terms selected in a semi-automated way were in fact tightly clustered in distributed semantic space.

Comparison of DDR scores to human annotations

We computed the point-biserial correlations between the 1000 annotated posts and their DDR scores to validate the accuracy of the DDR method. As indicated in Table S2, substantial correlations are observed across all the domains indicating high overlap between the predictions and human annotations.

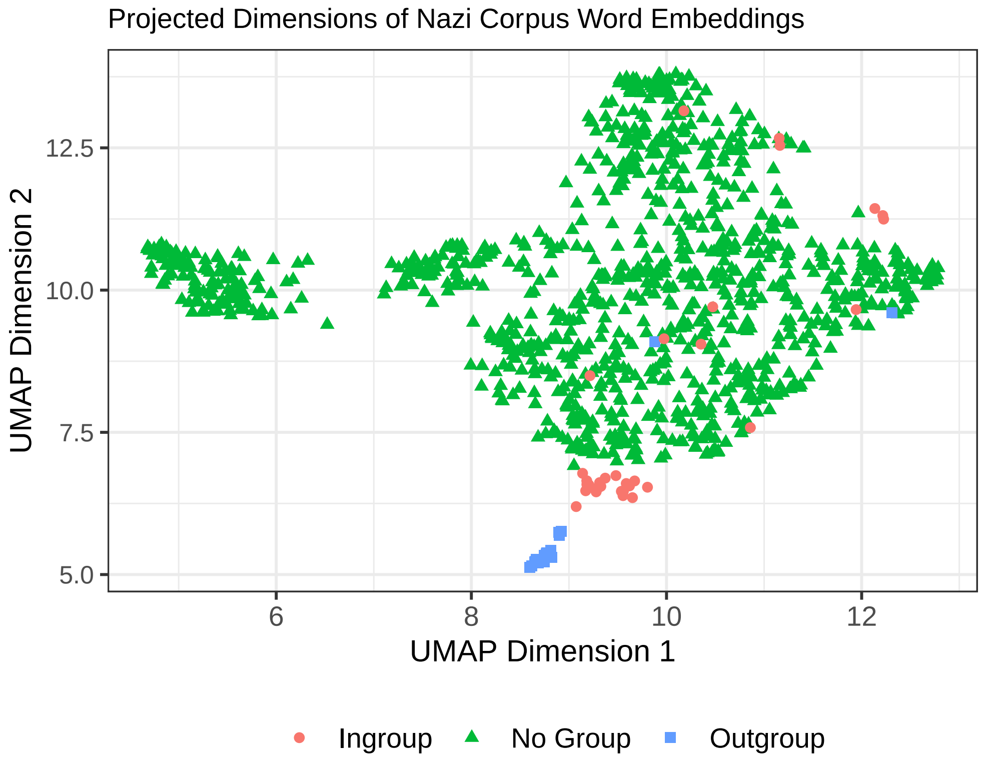

Figure S1

Visualization of the embeddings of all terms in the Nazi corpora, including the terms and phrases used to identify references to the ingroup and the outgroup. This figure clearly displays the tight clustering of both ingroup and outgroup terms, with a smaller number of outliers in both groups.

Table S2

Point-biserial correlation between annotated texts and DDR results across the five foundations

Moral foundation	Correlation
Care	0.151**
Fairness	0.075*
Loyalty	0.296**
Authority	0.198**
Purity	0.286**

Note: $^{*}p < 0.02$; $^{**}p < 0.001$.

Results

Analysis of Mein Kampf corpus

The main text reported in detail the results of our analysis on speeches and articles extracted from the Nazi propaganda archive, which combines both Mein Kampf and the Nazi Propaganda. In order to understand potential differences among the two corpora, a separate analysis of Mein Kampf and the Nazi propaganda was also conducted and is presented here. The primary difference between the Mein Kampf corpus and the Nazi propaganda corpus, aside from their difference in authorship and medium, was the relatively small size of the Mein Kampf corpus. This fact contributed to the lack of any significant findings regarding the difference in moral similarity among the different sentence types in Mein Kampf. However, the high-level findings in each corpus — in terms of the directionality in the effects between different sentence types — is observed in each of the two corpora.

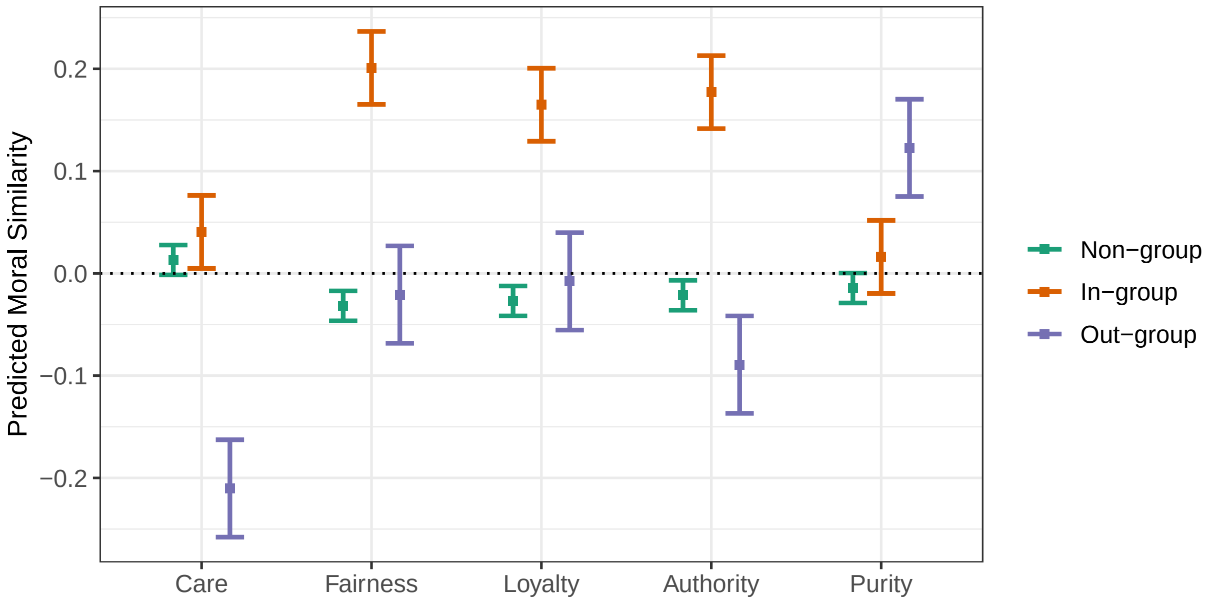

Figure S2
Estimated marginal means of moral similarity in the Nazi Propaganda, for combined factors of moral foundations category and document category. Predicted similarities are on a standardized scale. Error bars represent estimated 99% confidence intervals after Tukey-corrections for multiple comparisons.

We first report the findings based on the group-based analysis of the Nazi propaganda corpus; findings from the Mein Kampf corpus are provided next. For the mixed effects model of the effect of moral domain and sentence type on moral similarity, the intraclass-correlation coefficient for sentence-level varying intercepts was 0.644. There was a main effect of moral domain, $F(4, 155800.0) = 87.251$, $\eta_p^2 = 0.002$, $p < 0.001$, and of sentence type, $F(2, 38953.9) = 59.381$, $\eta_p^2 = 0.0007$, $p < 0.001$, with a significant interaction $F(8, 155800.0) = 140.310$, $\eta_p^2 = 0.007$, $p < 0.001$ (degrees of freedom approximated using Satterthwaite's method).

Post hoc analyses of interaction contrasts were conducted using Tukey's post-hoc test. Figure S2 contains the estimated marginal means from this model. Fairness similarity values for in-group sentences were significantly higher than Fairness similarity values for non-group (difference of 0.232) and out-group (difference of 0.222) sentences (ps < 0.0001). Similarly, Authority values were higher for in-group than out-group (0.266) and non-group (0.199) sentences, and Loyalty values were higher for in-group than out-group (0.156) and non-group (0.162) sentences (ps < 0.0001). This indicates that Fairness, Authority, and Loyalty concerns are generally invoked by Nazi speakers when discussing their own group. On the other side, Purity similarity values for out-group sentences were significantly higher than Purity similarity values for non-group (0.137, $p < 0.0001$) and in-group (0.107, $p < 0.0001$); inversely, Care similarity values for out-group sentences were *lower* than for non-group (0.223, $p < 0.0001$) and in-group (0.251, $p < 0.0001$) sentences.

For the mixed effects model of the effect of moral domain and sentence type on moral similarity for the Mein Kampf corpus, the intraclass-correlation coefficient for sentence-level varying intercepts was 0.644. No significant main effects or interactions from this model were significant. The estimated marginal means from this model are visualized in Figure S3. In-group morality similarities were higher than non-group similarities for fairness (difference of 0.122), Authority (0.102, $p = 0.0207$), and Loyalty (0.105, $p = 0.0163$). Though other relationships were not significant, the directionality of the effects

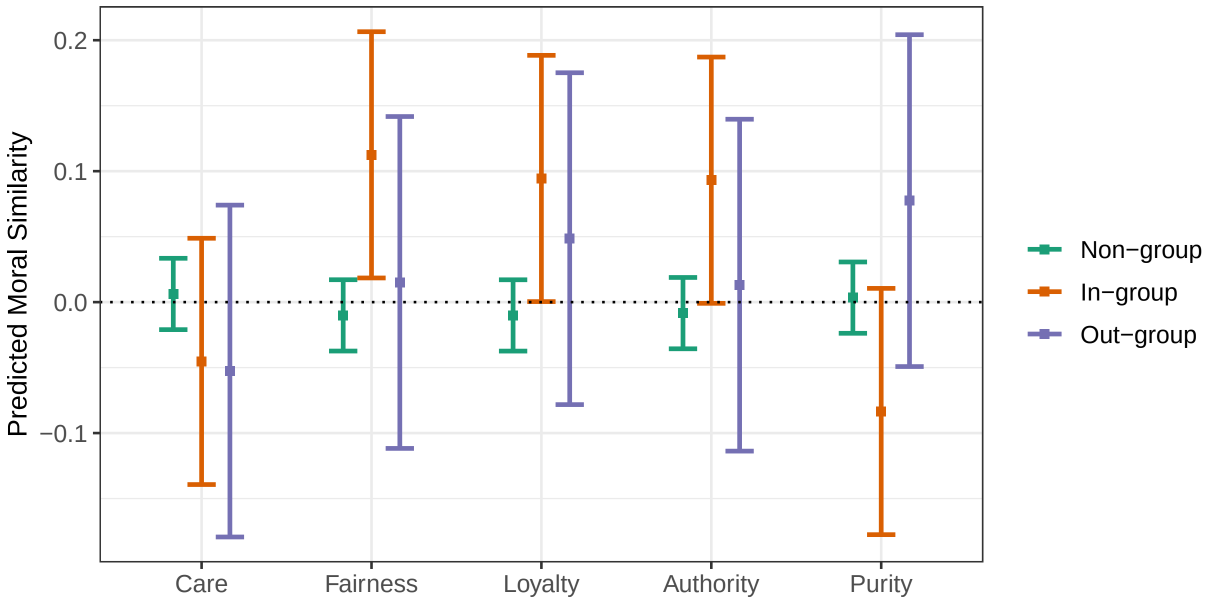

Figure S3

Estimated marginal means of the model of moral similarity in the Mein Kampf corpus.

mirrors closely the findings from the Nazi propaganda corpus reported in the main text. In particular, the moral similarity of out-group sentences is relatively high for Purity.

Comparison of Moral loading of Mein Kampf & Nazi Propaganda corpora with Wikipedia and King James Bible

A corpus of Wikipedia sentences from articles related to the Nazi texts (e.g., articles about Germans, the Holocaust, the Jews, etc.), was used as a “neutral” reference corpus, while a corpus containing the complete King James Bible[1] (Bible, 1989) was used as a “moral” reference corpus. The Bible text was preprocessed as corpora were above, and were split by individual verse (n = 24,971).

All Nazi texts were merged into a single sentence type. A model with varying intercepts for the sentence ID was fit, similar to the model above — with fixed effects and interaction for the moral category of a given similarity and the type of the sentence (Wikipedia, Bible, Nazi) — and had an ICC of 0.703. Significant effects of moral domain on sentences’ moral similarity, $F(4, 336100.0) = 780.179$, $\eta_p^2 = 0.009$, $p < 0.001$, for

[1] Full text accessed via Project Gutenberg, https://www.gutenberg.org/cache/epub/10/pg10.txt

sentence type, $F(2,\ 84018.0) = 9212.206$, $\eta_p^2 = 0.051$, $p < 0.001$, and interaction $F(8,\ 336100.0) = 3946.238$, $p < 0.001 = 0.084$.

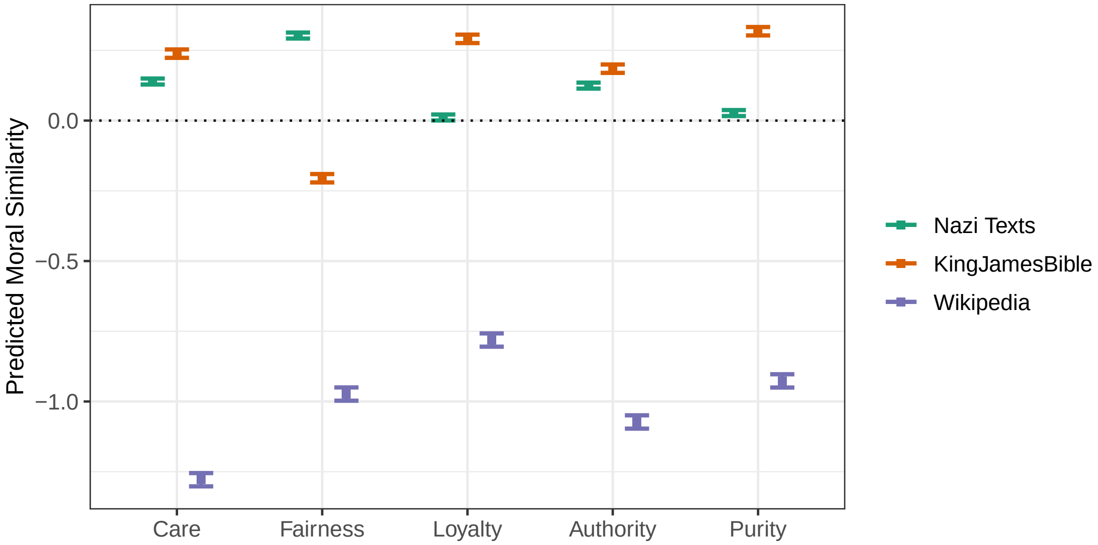

Figure S4

Estimated marginal means (z-scored) of each moral foundation and sentence type.

Estimated marginal means are visualized in Figure S4. Of particular concern is the fact that, for all moral domains, Wikipedia sentences had significantly lower moral loading than Nazi texts ($p < 0.0001$), indicating that all content analyzed in this study is significantly more moral than average text concerning a similar topic. Otherwise, all relationships displayed in Figure S4 were significant ($ps < 0.0001$).

Appendix B

Study 2: Cross-linguistic Analysis

Table S3

Moral foundations' seed words manually translated to the target languages

Language	Words
English	1. kindness 2. compassion 3. nurture 4. empathy 5. suffer 6. cruel 7. hurt 8. harm 9. fairness 10. equality 11. justice 12. rights 13. cheat 14. fraud 15. unfair 16. injustice 17. loyal 18. solidarity 19. patriot 20. fidelity 21. betray 22. treason 23. disloyal 24. traitor 25. authority 26. obey 27. respect 28. tradition 29. subversion 30. disobey 31. disrespect 32. chaos 33. purity 34. sanctity 35. sacred 36. wholesome 37. impurity 38. depravity 39. degradation 40. unnatural
Afrikaans	1. goedheid 2. medelye 3. koester 4. empatie 5. ly 6. wreed 7. seermaak 8. benadeel 9. regverdigheid 10. gelykheid 11. geregtigheid 12. regte 13. kul 14. bedrog 15. onregverdig 16. onreg 17. lojaal 18. solidariteit 19. patriot 20. getrouheid 21. verraai 22. verraad 23. dislojaal 24. verraaier 25. gesag 26. gehoorsaam 27. respek 28. tradisie 29. ondermyning 30. ongehoorsaam wees 31. gebrek aan respek 32. chaos 33. reinheid 34. onskendbaarheid 35. heilig 36. heilsaam 37. onreinheid 38. verdorwenheid 39. ontaarding 40. onnatuurlik
Arabic	10. الإنصاف 9. الضرر 8. الإيذاء 7. القسوة 6. المعاناة 5. التعاطف 4. الرعاية 3. الحنان 2. القلب طيبة 1.<br>18. مُخلص 17. الإجحاف 16. منصف غير 15. الاحتيال 14. الغش 13. الحقوق 12. العدالة 11. المساواة<br>27. الطاعة 26. السُلطة 25. غدّار 24. خائن 23. الخيانة 22. الغدر 21. الإخلاص 20. الوطنيّ 19. التضامن<br>36. مُقدّس 35. القُدسيّة 34. النقاء 33. الفوضى 32. الازدراء 31. العصيان 30. التخريب 29. العُرف 28. الاحترام<br>طبيعي غير 40. الانحلال 39. الأخلاقي الفساد 38. التلوث 37. مفيد
Bengali	1. উদারতা 2. সহানুভূতি 3. লালনপালন 4. সহমর্মিতা 5. ভোগা 6. নিষ্ঠুর 7. আঘাত 8. ক্ষতি 9. ন্যায়পরায়ণতা 10. সমতা 11. বিচার 12. অধিকার 13. প্রতারণা 14. জালিয়াতি 15. অন্যায় 16. অবিচার 17. অনুগত 18. সংহতি 19. দেশপ্রেমিক 20. বিশ্বস্ততা 21. বিশ্বাসঘাতকতাকরা 22. বিশ্বাসঘাতকতা 23. অবিশ্বস্ত 24. বিশ্বাসঘাতক 25. কর্তৃত্ব 26. মান্য 27. সম্মান 28. ঐতিহ্য 29. বিপর্যয় 30. অবাধ্য 31. অসম্মান 32. বিশৃঙ্খলা 33. বিশুদ্ধতা 34. পবিত্রতা 35. পবিত্র 36. স্বাস্থ্যকর 37. অপবিত্রতা 38. হীনতা 39. অধঃপতন 40. অপ্রাকৃত

Table S3

Moral foundations' seed words manually translated to the target languages

Language	Words
Bulgarian	1. доброта 2. състрадание 3. възпитаване 4. емпатия 5. страдание 6. жестокост 7. нараняване 8. ощетяване 9. честност 10. равенство 11. справедливост 12. права 13. измяна 14. измама 15. несправедливост 16. неправда 17. лоялност 18. солидарност 19. патриотизъм 20. вярност 21. предателство 22. измяна 23. нелоялност 24. изменничество 25. авторитетност 26. подчинение 27. уважение 28. традиция 29. подривна дейност 30. неподчинение 31. неуважение 32. хаос 33. чистота 34. святост 35. неприкосновеност 36. цялост 37. нечистота 38. поквара 39. деградация 40. неестественост
Dutch	1. vriendelijkheid 2. medeleven 3. zorgzaam 4. empathie 5. lijden 6. wreed 7. leed (n), pijn doen (v) 8. beschadigen 9. eerlijkheid 10. gelijkheid 11. rechtvaardigheid 12. rechten 13. bedriegen 14. fraude 15. niet eerlijk 16. onrechtvaardig 17. loyaal 18. saamhorigheid 19. patriot 20. trouw 21. verraden 22. verraad 23. niet loyaal 24. verrader 25. authoriteit 26. gehoorzamen 27. respect (n), respecteren (v) 28. traditie 29. subversie 30. niet gehoorzamen 31. respectloos (n), niet respecteren (v) 32. chaos 33. zuiverheid 34. heiligheid 35. heilig 36. gezond 37. onzuiverheid 38. verdorvenheid 39. degradatie 40. onnatuurlijk
French	1. gentillesse 2. compassion 3. nourrir, encourager, alimenter 4. empathie 5. souffrir 6. cruel 7. blesser 8. (v) nuire, blesser, endommager / (n) préjudice, tort, mal 9. équité 10. égalité 11. justice 12. droits 13. tricher 14. fraude 15. injuste 16. injustice 17. loyal 18. solidarité 19. patriote 20. fidélité 21. trahir 22. trahison 23. déloyal 24. traître 25. autorité 26. obéir 27. (v) respecter / (n) respect 28. tradition 29. subversion 30. désobéir 31. (v) manquer de respect / (n) manque de respect 32. chaos 33. pureté 34. sainteté 35. sacré 36. sain 37. impureté 38. dépravation 39. dégradation 40. contre nature
German	1. Liebenswürdigkeit 2. Mitgefühl 3. erziehen (v), Erziehung (n) 4. Einfühlungsvermögen 5. leiden 6. grausam 7. wehtun (v), verletzt (adj./p.p.) 8. beschädigen (v), Schaden (n) 9. Gerechtigkeit 10. Gleichberechtigung 11. Recht 12. Rechte 13. täuschen (v), Täuschung (n) 14. Betrug 15. ungerecht 16. Unrecht 17. loyal 18. Solidarität 19. Patriot 20. Treue 21. betrügen 22. Verrat 23. illoyal 24. Verräter 25. Autorität 26. gehorchen 27. respektieren (v), Respekt (n) 28. Tradition 29. Umsturz 30. missachten 31. nicht respektieren (v), Nichtachtung (n) 32. Chaos 33. Reinheit 34. Unantastbarkeit 35. heilig 36. heilsam 37. Unreinheit 38. Schlechtigkeit 39. Zerfall 40. unnatürlich

Table S3

Moral foundations' seed words manually translated to the target languages

Language	Words
Greek	1. καλοσύνη 2. συμπόνια 3. ανατρέφω / ανατροφή 4. ενσυναίσθηση 5. υποφέρω 6. σκληρός 7. πληγώνω / πληγωμένος 8. βλάπτω / βλάβη 9. νομιμότητα, δικαιοσύνη 10. ισότητα 11. δικαιοσύνη 12. δικαιώματα 13. ατιμία 14. απάτη 15. άδικος 16. αδικία 17. πιστός 18. αλληλεγγύη 19. πατριώτης 20. πίστη / αφοσίωση 21. προδίδω 22. προδοσία 23. άπιστος 24. προδότης 25. εξουσία 26. υπακούω 27. σέβομαι / σεβασμός 28. παράδοση 29. ανατροπή 30. απειθώ 31. προσβάλλω / ασέβεια 32. χάος 33. αγνότητα 34. ιερότητα 35. ιερός 36. ευεργετικός, ψυχωφελής 37. ακαθαρσία, ανηθικότητα 38. ανηθικότητα, ακολασία 39. υποβάθμιση, εξαχρείωση 40. αφύσικος
Hindi	1. दयालुता 2. करुणा 3. पालन-पोषण करना 4. सहानुभूति 5. कष्ट सहना 6. निर्दयी 7. चोट पहुंचाना 8. नुकसान 9. निष्पक्षता 10. समानता 11. न्याय 12. अधिकार 13. धोखा 14. कपट 15. अनुचित 16. अन्याय 17. निष्ठावान 18. एकजुटता 19. देश-भक्त 20. सत्य निष्ठा 21. धोखा देना 22. राजद्रोह 23. विश्वासघाती 24. गद्दार 25. प्राधिकरण 26. आज्ञा का पालन 27. सम्मान 28. परंपरा 29. विनाश 30. आज्ञा न मानना 31. अनादर 32. अव्यवस्था 33. पवित्रता 34. शुद्धता 35. धार्मिक 36. स्वास्थ्यकर 37. अपवित्रता 38. दुराचार 39. दर्जा घटाना 40. अस्वाभाविक
Indonesian	1. kebaikan hati, kemurahan hati 2. kasih sayang 3. memelihara 4. empati 5. menderita 6. kejam 7. melukai, menyakiti 8. merugikan 9. keadilan, kejujuran 10. persamaan, ekualitas 11. keadilan 12. hak 13. menipu, memfitnah 14. penipu, penipuan 15. tidak adil 16. ketidakadilan 17. setia 18. solidaritas 19. patriot 20. kestiaan, loyalitas 21. mengkhianati, memfitnah 22. pengkhianatan 23. tidak setia 24. pengkhianat 25. otoritas, yang berwenang 26. mematuhi, menaati 27. menghormati 28. tradisi, adat 29. subversi 30. tidak mematuhi, 31. tidak menghormati 32. kekacauan, kekacau-balauan 33. kemurnian, kesucian 34. kesucian 35. Suci, kudus, keramat 36. baik, sehat 37. ketidakmurnian 38. kerusakan moral 39. degradasi, 40. tidak wajar
Italian	1. gentilezza 2. comprensione 3. educazione 4. empatia 5. soffrire 6. crudele 7. ferire 8. sofferenza 9. correttezza 10. uguaglianza 11. giustizia 12. diritti 13. imbrogliare 14. truffa 15. ingiusto 16. ingiustizia 17. leale 18. solidarietà 19. patriota 20. fedeltà 21. tradire 22. tradimento 23. sleale 24. traditore 25. autorità 26. obbedire 27. rispetto 28. tradizione 29. sovversione 30. disobbedire 31. mancanza di rispetto 32. caos 33. purezza 34. santità 35. sacro 36. sano 37. impurità 38. corruzione morale 39. degradazione 40. innaturale

Table S3

Moral foundations' seed words manually translated to the target languages

Language	Words
Japanese	1. 親切 2. 思いやり 3. 育成 4. 共感 5. 苦しむ 6. 残酷 7. 傷つける 8. 害する 9. 公平さ 10. 平等 11. 正義 12. 権利 13. 騙す 14. 詐欺 15. 不公平 16. 不正 17. 忠実 18. 連帯 19. 愛国者 20. 忠誠 21. 裏切り 22. 反逆 23. 不忠実 24. 反逆者 25. 権威 26. 従う 27. 尊敬 28. 伝統 29. 転覆 30. 不服従 31. 軽蔑 32. 大混乱 33. 純粋さ 34. 尊厳 35. 神聖な 36. 健全な 37. 不純 38. 堕落 39. 劣化 40. 不自然
Korean	1. 친절 2. 연민 3. 보살핌 4. 공감 5. 괴로움 6. 잔혹한 7. 상처 8. 피해 9. 공정 10. 평등 11. 정의 12. 권리 13. 기만 14. 사기 15. 부당한 16. 불의 17. 충실한 18. 결속 19. 애국자 20. 신의 21. 배신 22. 반역 23. 불충 24. 반역자 25. 권위 26. 복종 27. 존경 28. 전통 29. 전복 30. 불복종 31. 무례 32. 혼란 33. 순결 34. 존엄성 35. 성스러운 36. 건전한 37. 불결 38. 타락 39. 비하 40. 부자연스러운
Persian	1. مهربانی 2. دلسوزی 3. پرورش دادن 4. یکدلی 5. رنج بردن 6. ظالم 7. آسیب 8. صدمه 9. انصاف 10. برابری 11. عدالت 12. حقوق 13. تقلب 14. کلاهبرداری 15. غیرمنصفانه 16. بی‌عدالتی 17. وفادار 18. همبستگی 19. میهن‌پرست 20. وفاداری 21. خیانت کردن 22. خیانت 23. بی‌وفا 24. خائن 25. اختیار 26. اطاعت کردن 27. احترام 28. سنت 29. براندازی 30. نافرمانی 31. بی‌احترامی 32. هرج‌ومرج 33. خلوص 34. قداست 35. مقدس 36. سالم 37. ناخالصی 38. هرزگی 39. تنزل 40. غیرطبیعی
Polish	1. życzliwość 2. współczucie 3. pielęgnowanie 4. empatia 5. cierpieć 6. okrutny 7. skrzywdzony, ranny, uraz 8. krzywda 9. uczciwość 10. równość 11. sprawiedliwość 12. prawa 13. oszukać 14. oszustwo 15. niesprawiedliwy 16. niesprawiedliwość 17. lojalny 18. solidarność 19. patriota 20. wierność 21. zdradzić 22. zdrada 23. nielojalny 24. zdrajca 25. autorytet 26. posłuszeństwo 27. szacunek 28. tradycja 29. subwersja, przewrót, działalność wywrotowa 30. nieposłuszeństwo 31. znieważenie 32. chaos 33. czystość 34. świętość 35. święty 36. zdrowy 37. nieczystość 38. deprawacja 39. degradacja 40. nienaturalny
Portuguese	1. bondade 2. compaixão 3. cultivar 4. empatia 5. sofrer 6. cruel 7. machucar, dor 8. prejudicar, dano 9. imparcialidade 10. igualdade 11. justiça 12. direitos 13. enganar, trapaça 14. fraude 15. injusto 16. injustiça 17. leal 18. solidariedade 19. patriota 20. fidelidade 21. trair 22. traição 23. desleal 24. traidor 25. autoridade 26. obedecer 27. respeitar, respeito 28. tradição 29. subversão 30. desobedecer 31. desrespeitar, desrespeito 32. caos 33. pureza 34. santidade 35. sagrado 36. saudável 37. impureza 38. depravação 39. degradação 40. anormal, artificial

Table S3

Moral foundations' seed words manually translated to the target languages

Language	Words
Romanian	1. bunătate 2. compasiune 3. a încuraja 4. empatie 5. a suferi 6. crud 7. ofensă 8. vătămare 9. corectitudine 10. egalitate 11. dreptate 12. drepturi 13. înşelăciune 14. escrocherie 15. necinstit 16. nedreptate 17. loial 18. solidaritate 19. patriot 20. loialitate 21. a trăda 22. trădare 23. neloial 24. trădător 25. autoritate 26. a se supune 27. respect 28. tradiție 29. subversiune 30. a nu se supune 31. lipsă de respect 32. haos 33. puritate 34. sfinţenie 35. sacru 36. sănătos 37. necuviință 38. depravare 39. degradare 40. nenatural
Russian	1. доброта 2. сочувствие 3. забота 4. эмпатия 5. страдать 6. жестокий 7. страдания 8. вред 9. справедливость 10. равноправие 11. правосудие 12. права 13. обман 14. мошенничество 15. несправедливый 16. проявление несправедливости 17. верный 18. солидарность 19. патриот 20. верность 21. предавать 22. государственная измена 23. неверный 24. изменник 25. авторитет 26. слушаться 27. уважение 28. традиция 29. свержение 30. не слушаться 31. неуважение 32. хаос 33. чистота 34. священность 35. святой 36. благоприятный 37. загрязнение 38. порочность 39. ухудшение 40. неестественный
Spanish	1. amabilidad 2. compasión 3. crianza 4. empatía 5. sufrir 6. cruel 7. lastimar 8. daño 9. imparcialidad 10. igualdad 11. justicia 12. derechos 13. engañar 14. fraude 15. injusto 16. injusticia 17. leal 18. solidaridad 19. patriota 20. fidelidad 21. traicionar 22. traición 23. desleal 24. traidor 25. autoridad 26. obedecer 27. respeto 28. tradición 29. subversión 30. desobedecer 31. falta de respeto 32. caos 33. pureza 34. santidad 35. sagrado 36. íntegro 37. impureza 38. inmoralidad 39. humillación 40. forzado
Swedish	1. vänlighet 2. medmänsklighet 3. (n) uppfostran\n(v) fostra 4. empati 5. lida 6. grym 7. (n) smärta\n(v) skada 8. skada 9. rimlighet 10. jämlikhet 11. rättvisa 12. rättigheter 13. fuska 14. bedrägeri 15. orättvis 16. orättvisa 17. lojal 18. solidaritet 19. patriot 20. trohet 21. förråda 22. förräderi 23. illojal 24. förrädare 25. myndighet 26. lyda 27. respektera 28. tradition 29. omstörtning 30. inte lyda 31. (n) respektlöshet\n(v) inte respektera 32. kaos 33. renhet 34. helighet 35. helig 36. hälsosam 37. orenhet 38. demoralisering 39. försämring 40. onaturlig

Table S3

Moral foundations' seed words manually translated to the target languages

Language	Words
Tamil	1. இரக்கம் 2. அன்பு 3.போஷி 4. அனுதாபம் 5. பாதிப்பு 6. கொடூரம் 7. காயப்படுத்து 8. தீங்கு 9. நியாயம் 10. சமத்துவம் 11. நீதி 12. உரிமைகள் 13. ஏமாற்று 14. மோசடி 15. நியாயமற்ற 16. அநீதி 17. விசுவாசம் 18. ஒற்றுமை 19. தேசபக்தர் 20. நம்பிகத்தன்மை 21. காட்டிக்கொடு 22. துரோகம் 23. விசுவாசமற்ற 24. துரோகி 25. அதிகாரம் 26. கீழ்ப்படி 27. மரிதாயை 28. பாரம்பரியம் 29. அடிபணிதல் 30. கீழ்ப்படியாமை 31. அவமரியாதை 32. குழப்பம் 33. தூய்மை 34. புனிதத்தன்மை 35. புனிதமான 36. முழுமையான 37. அசுத்தம் 38. சீரழிவு 39. சிதைவு 40. இயற்கைக்கு மாறான
Turkish	1. nezaket 2. şefkat 3. beslemek 4. empati 5. acı çekmek 6. zalim 7. incitmek\nacımak 8. zarar vermek\nzarar 9. adillik 10. eşitlik 11. adalet 12. haklar 13. aldatmak 14. dolandırmak\nsahtekarlık\nsahtekar 15. haksız 16. adaletsizlik 17. sadık 18. dayanışma 19. vatansever 20. sadakat 21. ihanet etmek 22. hainlik 23. sadakatsiz 24. vatan haini 25. otorite\nyetkili 26. itaat etmek 27. saygı duymak 28. gelenek 29. yıkıcılık 30. itaatsizlik etmek 31. saygısızlık etmek 32. kaos 33. saflık 34. kutsallık 35. kutsal 36. erdemli\ngüvenilir 37. kirlilik, katışıklık 38. ahlak bozukluğu 39. yozlaşma\nbozulma 40. doğaya aykırı\ndoğal olmayan\nyapay
Urdu	9. نقصان 8. مجروح 7. ظالم 6. سہنا 5. ہمدردی 4. پرورش 3. شفقت 2. رحمدلی 1.<br>غیر 15. فراڈ 14. دھوکہ 13. حقوق 12. انصاف 11. مساوات 10. پسندی انصاف<br>21. مخلص 20. وطن محب 19. یکجہتی 18. وفادار 17. ناانصافی 16. منصفانہ<br>28. احترام 27. اطاعت 26. اتھارٹی 25. غدار 24. وفا بے 23. غداری 22. خیانت<br>تقدس 34. پاکیزگی 33. افراتفری 32. عزتی بے 31. نافرمانی 30. بغاوت 29. روایت<br>فطری غیر 40. تنزلی 39. بدحالی 38. نجاست 37. بخش صحت 36. مقدس 35.
Vietnamese	1. lòng tốt 2. lòng thương cảm 3. sự nuôi dưỡng 4. sự đồng cảm 5. đau khổ 6. hung ác 7. xúc phạm 8. gây hại 9. công bằng 10. bình đẳng 11. công chính 12. quyền lợi 13. gian lận 14. lừa đảo 15. không công bằng 16. bất công 17. trung thành 18. đoàn kết 19. người yêu nước 20. trung thực 21. phản bội 22. bội tín 23. không trung thành 24. kẻ phản bội 25. chính quyền 26. tuân theo 27. tôn trọng 28. truyền thống 29. lật đổ 30. không tuân theo 31. không tôn trọng 32. hỗn loạn 33. trong sạch 34. thiêng liêng 35. linh thiêng 36. lành mạnh 37. không trong sạch 38. trụy lạc 39. thoái hóa 40. trái với tự nhiên

Relation between hate and other linguistic categories

In the main text, we reported results on the moral loading of hateful terms across languages, finding that hateful terms loaded most heavily onto Loyalty language in comparison to other moral domains. However, this result lacks context with respect to the moral loading of basic linguistic categories, as the moral loading of hateful terms may reflect a basic trend regarding the moral loading of word types across languages. To ensure that our findings were not reflective of generic semantic trends, we computed the moral loadings of all content-word categories (N = 48) defined in the Linguistic Inquiry and Word Count (LIWC; Pennebaker et al., 2015) for the languages used in our study. We sampled 15 words for each category and relied on the openly available Google translate API [2] to translate them to non-English languages. Given that this approach fundamentally introduces noise due to the lack of translation by native speakers, we adopted a back-translation approach to decrease the level of noise in the translations.

To yield a single (average) similarity score per MFD/LIWC category and language we averaged the cosine similarities of each hateful term with words from a given MFD/LIWC category per language. We standardized the similarity scores of all the MFD and LIWC categories for each language. The results of our comparison are visualized in Figure S5.

[2] https://cloud.google.com/translate/docs/reference/libraries/v2/python

LIWC/MFD Category	Afrikaans	Arabic	Bengali	Bulgarian	Dutch	English	Finnish	French	German	Greek	Hindi	Indonesian	Italian	Japanese	Korean	Persian	Polish	Portuguese	Romanian	Russian	Spanish	Swedish	Turkish	Urdu	Vietnamese
Female references	-0.05	0.16	0.46	0.25	0.47	0.92	0.89	0.31	1.23	0.40	0.11	0.57	0.67	0.41	-0.07	-0.01	0.56	0.57	0.56	0.50	0.73	0.59	0.56	-0.53	-0.22
Comparisons	0.28	0.32	-0.24	0.65	0.23	-0.12	-0.56	0.63	0.12	-0.14	-0.38	0.74	0.82	0.61	2.99	0.54	-0.44	0.13	1.34	0.18	0.65	0.24	-0.11	0.41	0.21
Anger	0.44	0.04	0.65	0.42	0.69	0.63	0.85	0.27	0.67	0.15	-0.19	0.25	0.80	-0.34	-0.41	0.24	0.49	0.48	0.83	0.12	0.65	0.49	0.59	-0.14	0.15
Biological processes	0.23	0.31	0.21	0.44	0.30	0.45	0.63	0.34	0.40	0.12	0.26	0.13	0.36	-0.16	0.21	0.10	0.32	0.66	0.25	0.09	0.96	0.36	-0.22	0.58	0.01
Home	-0.07	0.66	0.31	0.66	0.18	-0.09	0.31	0.30	0.31	0.17	0.31	0.51	0.49	-0.22	-0.49	0.37	0.29	0.54	0.65	0.55	-0.19	-0.39	0.37	0.32	0.00
Loyalty	0.06	-0.15	-0.04	0.13	0.84	0.26	0.46	0.24	0.47	0.23	0.09	0.49	0.26	-1.22	-1.55	0.28	0.75	0.37	0.39	0.62	0.40	0.36	1.64	-0.04	0.48
Common adjectives	-0.25	-0.11	-0.43	0.23	-0.32	0.07	-0.04	0.65	0.02	0.53	-0.27	0.43	0.75	0.40	1.85	0.43	0.07	-0.62	0.08	0.21	0.52	0.38	0.08	0.25	0.02
Negative emotion	0.02	-0.03	-0.12	-0.02	0.71	0.12	0.29	0.20	0.19	0.02	-0.14	0.27	0.07	-0.28	0.62	0.27	0.30	0.83	0.12	-0.08	0.60	0.48	0.47	-0.08	-0.04
Tentative	0.40	0.07	0.71	0.56	0.30	0.06	-0.05	0.07	-0.12	0.15	0.30	0.37	-0.07	0.20	0.53	0.42	0.10	0.29	0.03	-0.15	-0.18	0.17	-0.29	0.92	-0.16
Ingestion	0.44	-0.07	0.13	0.15	0.72	0.16	0.46	0.22	0.57	0.17	-0.26	0.35	0.23	-0.31	-0.39	-0.02	0.10	0.36	0.45	0.44	0.59	0.22	0.16	-0.32	-0.05
Religion	0.10	-0.14	0.41	0.21	-0.18	0.21	0.43	0.01	0.11	0.41	0.23	-0.42	0.14	-0.29	-0.33	0.22	1.02	0.45	0.76	0.26	0.18	0.19	0.12	0.04	-0.18
Social processes	0.26	0.15	0.39	-0.39	0.18	0.50	0.38	0.51	0.56	0.07	-0.03	0.21	0.09	-0.18	0.17	-0.13	0.25	0.26	-0.07	0.01	0.08	0.09	0.51	0.10	-0.04
Positive emotion	0.18	-0.02	0.68	-0.06	0.12	-0.17	-0.03	0.31	0.32	-0.03	0.21	-0.24	0.24	0.23	0.11	-0.19	0.22	0.16	0.49	-0.12	0.29	-0.04	0.03	0.12	0.32
Body	0.17	0.22	-0.07	0.07	0.03	0.44	0.90	0.37	0.63	0.33	-0.02	-0.23	0.53	-0.64	-0.76	-0.17	0.59	0.01	-0.42	0.58	1.00	-0.18	0.38	-1.04	-0.01
Male references	0.09	0.29	0.06	-0.35	0.51	0.62	0.67	0.05	0.76	-0.12	-0.29	-0.21	0.28	0.07	-1.14	0.12	0.38	-0.06	0.19	0.29	0.16	0.11	0.34	-0.14	-0.36
Hear	0.03	-0.21	-0.39	-0.09	-0.15	-0.15	0.28	0.12	0.37	-0.17	-0.06	0.32	0.07	-0.21	0.48	-0.06	-0.04	0.26	0.22	-0.02	0.32	0.39	0.10	0.86	0.02
Perceptual processes	0.10	0.05	0.35	-0.04	-0.15	0.05	-0.02	0.41	0.08	-0.06	0.01	-0.10	0.43	-0.38	0.63	0.10	-0.09	0.14	-0.16	0.00	0.11	0.32	0.01	0.29	0.12
Certainty	0.18	0.18	-0.07	0.15	0.39	-0.09	-0.04	0.24	0.09	-0.12	0.16	0.37	-0.10	0.45	-0.65	-0.04	0.10	0.14	0.09	0.00	-0.12	0.50	-0.34	0.57	0.12
Sadness	0.35	-0.18	-0.47	-0.25	0.42	-0.16	0.39	0.30	0.20	0.06	-0.09	-0.25	0.57	0.05	-0.69	0.40	0.41	0.27	0.04	0.01	0.62	-0.11	-0.11	0.24	-0.14
Family	0.26	0.08	-0.32	-0.30	0.00	0.67	0.72	-0.11	0.46	-0.62	-0.29	-0.32	-0.11	0.49	-0.85	0.11	0.40	0.33	0.47	0.46	-0.03	-0.26	0.41	-0.16	-0.13
Sexual	-0.07	0.78	0.33	-0.03	-0.30	0.23	0.13	-0.17	0.27	0.61	-0.05	-1.01	0.21	-0.12	-0.20	-0.18	-0.09	0.24	-0.41	0.63	0.04	-0.55	-0.06	0.77	0.12
Power	0.09	0.22	0.11	0.20	-0.27	-0.02	0.05	-0.12	-0.10	-0.14	0.12	-0.44	-0.12	0.27	1.12	-0.25	-0.28	-0.02	0.04	0.07	0.11	0.10	0.08	0.01	-0.12
Future focus	0.13	-0.12	0.75	0.03	0.14	-0.30	-0.16	-0.19	-0.05	0.02	0.14	0.72	-0.27	0.51	0.41	0.16	-0.41	-0.44	-0.10	-0.19	-0.19	0.36	-0.47	-0.18	-0.01
Affective processes	-0.08	-0.07	0.27	-0.02	0.01	-0.15	-0.07	-0.01	-0.07	0.16	0.28	0.22	-0.18	-0.19	0.07	0.17	-0.05	-0.24	0.05	-0.31	-0.12	-0.09	0.13	0.65	-0.12
Authority	0.52	-1.00	-0.59	0.00	0.27	-0.08	0.17	-0.02	-0.49	0.42	-0.27	0.50	-0.34	-0.40	-1.28	-0.23	0.37	-0.19	-0.38	0.02	0.37	1.26	0.73	-0.23	0.34
Numbers	-0.04	0.20	-0.50	-0.13	0.24	0.09	-0.05	0.00	-0.10	-0.26	-0.50	0.14	0.23	0.37	0.12	-0.49	0.21	0.48	0.47	-0.29	-0.33	0.49	-0.37	-0.66	-0.31
Friends	0.12	0.22	-0.49	-0.34	-0.16	0.62	0.71	-0.28	0.03	-0.35	-0.02	0.04	0.04	-0.04	0.29	-0.22	-0.31	-0.27	0.10	-0.02	-0.11	-0.12	0.12	-0.79	0.16
Leisure	0.07	0.03	-0.28	0.29	-0.24	0.04	0.06	0.09	0.12	0.07	-0.55	-0.05	-0.24	0.47	-0.73	-0.04	0.22	0.08	0.08	0.32	-0.04	0.20	-0.32	-0.53	-0.26
Cognitive processes	-0.09	0.16	-0.04	-0.05	-0.28	-0.38	-0.85	0.01	-0.61	-0.09	0.59	-0.10	-0.11	0.81	0.57	0.19	-0.22	-0.40	-0.57	0.06	-0.44	0.45	-0.24	0.39	0.02
Past focus	-0.28	-0.36	0.38	0.11	-0.21	-0.28	-0.45	-0.32	-0.33	0.03	0.00	-0.18	-0.21	0.55	0.04	0.26	-0.17	-0.14	-0.15	-0.58	-0.16	0.21	-0.28	1.68	-0.48
Care	-0.23	-0.85	-0.56	-0.24	0.35	0.16	0.74	0.09	0.21	0.53	0.51	-0.20	0.12	-1.07	-0.50	-0.10	0.28	-0.03	0.40	-0.31	0.02	0.04	0.62	-1.34	0.07
Health	0.14	0.15	0.35	-0.01	-0.40	-0.04	-0.20	-0.33	-0.12	0.40	-0.49	-0.25	-0.25	0.00	0.41	-0.25	-0.10	-0.22	-0.17	-0.13	0.88	-0.21	-0.56	0.17	-0.17
Differentiation	-0.38	0.25	-0.49	-0.04	-0.10	-0.11	-0.50	-0.45	-0.56	0.45	-0.17	0.21	-0.64	0.81	1.57	0.00	-0.01	-0.20	-0.18	-0.35	-1.28	0.62	-0.04	0.18	-0.01
Anxiety	0.06	-0.36	-0.27	-0.35	0.19	-0.33	-0.23	-0.12	-0.19	-0.24	0.26	-0.14	-0.06	-0.07	-0.24	0.16	-0.21	0.08	-0.33	-0.12	-0.02	0.20	-0.01	0.42	0.39
Fairness	-0.40	-0.70	-0.44	-0.14	0.73	0.07	0.28	0.09	0.37	-0.44	-0.53	0.41	0.05	-1.03	-1.06	-0.53	0.72	0.15	-0.20	0.32	0.13	-0.03	0.86	-0.47	0.19
Common verbs	0.36	-0.16	0.00	-0.27	-0.07	0.00	-0.36	-0.12	0.20	0.07	-0.09	-0.42	0.08	0.08	0.16	0.02	-0.10	0.02	-0.15	-0.27	-0.32	0.08	-0.53	0.13	0.04
Purity	0.26	-1.25	-0.14	0.15	-0.09	-0.16	0.24	0.24	-0.06	-0.13	0.18	0.29	0.27	-1.02	-1.32	-0.61	0.31	0.42	0.14	-0.15	0.22	-0.18	0.41	-0.08	0.33
Feel	-0.17	-0.28	-0.10	0.05	-0.14	-0.05	-0.14	0.06	-0.09	0.01	0.07	0.07	0.17	0.01	-0.14	-0.09	-0.16	0.03	-0.13	0.02	0.15	-0.28	-0.23	-1.12	0.00
Affiliation	0.02	-0.20	-0.02	-0.11	-0.10	-0.06	-0.11	-0.24	-0.09	-0.03	0.07	-0.12	-0.36	0.33	-0.08	-0.12	-0.30	-0.47	0.02	-0.31	-0.22	0.02	-0.09	-0.16	0.02
Death	-0.09	0.27	0.67	-0.38	0.14	-0.20	-0.09	-0.19	-0.23	-0.05	0.17	-0.34	-0.49	0.23	0.37	0.35	-0.53	-0.46	0.10	-0.02	-0.58	-0.98	-0.09	-0.58	0.03
Swear words	-0.24	0.66	0.38	-0.07	-0.82	0.66	-0.25	-0.36	0.06	0.26	0.67	-1.36	-0.04	0.59	-0.02	-0.37	-0.14	-0.07	-0.63	0.54	0.16	-1.16	-0.25	-1.27	-0.14
Money	-0.14	-0.17	0.32	0.02	-0.41	-0.12	-0.25	-0.25	-0.11	-0.40	-0.07	-0.16	-0.16	0.24	-0.02	-0.40	-0.27	-0.04	0.03	-0.38	-0.07	-0.43	-0.14	-0.31	0.12
Causation	-0.22	-0.07	0.43	0.35	-0.22	-0.37	-0.94	-0.30	-0.56	-0.20	0.18	-0.14	-0.38	-0.33	0.92	0.06	0.00	-0.23	-0.48	-0.16	-0.76	-0.49	-0.29	0.66	-0.27
Time	-0.33	0.17	-0.06	-0.16	-0.03	-0.13	-0.55	-0.27	-0.77	-0.06	0.11	0.12	-0.64	0.82	-0.02	-0.07	-0.34	-0.59	-0.34	-0.20	-0.13	-0.17	-0.46	-0.24	0.10
Risk	-0.35	-0.33	-0.02	-0.23	-0.33	-0.17	-0.25	-0.17	-0.47	-0.36	0.53	0.32	-0.28	-0.23	-0.07	-0.19	-0.17	-0.15	-0.21	-0.48	-0.14	0.02	-0.30	-0.34	-0.01
Drives	0.15	-0.05	-0.49	-0.27	-0.24	-0.21	-0.09	-0.03	-0.35	-0.42	-0.43	-0.30	-0.15	0.04	-1.01	-0.03	0.22	-0.36	-0.12	-0.07	-0.48	-0.32	0.05	-0.06	0.15
Insight	-0.32	-0.25	0.18	-0.22	-0.25	-0.78	-0.81	-0.50	-0.62	-0.21	1.11	-0.35	-0.75	-0.22	1.30	0.43	-1.04	-0.10	-0.50	-0.56	-0.71	-0.06	-0.38	0.70	-0.04
See	-0.29	-0.19	0.04	-0.13	-0.31	-0.43	-0.35	-0.30	-0.29	-0.26	0.09	0.09	-0.56	0.09	0.07	-0.13	-0.16	-0.31	-0.70	-0.07	-0.43	-0.60	-0.56	0.30	0.18
Achievement	-0.45	-0.06	-0.23	-0.20	-0.58	-0.45	-0.67	-0.06	-0.53	-0.29	0.16	-0.23	-0.52	0.34	0.26	-0.01	-0.72	-0.79	-0.47	-0.20	-0.77	-0.32	-0.29	0.31	0.22
Work	-0.13	0.38	-0.29	0.26	-0.66	-0.32	-0.45	-0.82	-0.63	-0.03	-0.34	-0.22	-0.35	0.21	-0.03	0.11	-0.49	-0.48	-0.54	0.09	-0.21	-0.67	-0.58	-0.19	-0.21
Motion	-0.17	-0.21	-0.25	0.06	-0.42	-0.47	-0.71	-0.09	-0.21	-0.18	-0.37	-0.20	-0.01	-0.27	-0.27	-0.17	-0.86	-0.04	-0.01	-0.10	-0.78	-0.31	-0.18	-0.17	-0.23
Space	-0.32	-0.09	-0.45	-0.23	-0.28	-0.05	-0.26	-0.12	-0.37	-0.02	-0.62	0.02	-0.11	-0.21	-0.47	-0.37	-0.21	-0.41	-0.38	-0.35	-0.52	-0.64	-0.41	-0.80	-0.26
Reward	-0.371	-0.114	-0.675	-0.300	-0.474	-0.543	-0.506	-0.197	-0.679	-0.394	-0.430	-0.201	-0.441	-0.262	-0.458	-0.049	-0.747	-0.454	-0.638	-0.378	-0.617	-0.347	-0.564	0.912	0.006

Figure S5

Standardized (within language) LIWC and MFD categories' loadings of hateful terms across languages. LIWC and MFD categories are ordered by the average loading across the 25 languages.

Appendix C

Study 3: Large-scale Social Media Analysis

Samples of moral hate speech

Table S4

Examples of hate-based rhetoric (as agreed to by a majority of annotators in the Gab Hate Corpus) that are also annotated as containing moral foundations content. Three examples are given for each of the five foundations.

Care	If you are right-wing and pay to send your daughter to college, you are retarded. Woman are submissive and much more prone to peer pressure. They are not equipped (NAWALT) to handle life in a re-education center. By sending her to college, you are destroying her future and increasing the risk she'll die in childless misery
	Islam causes overpopulation... by enslaving women...
	UK: Muslims forced woman to drink alcohol, then one raped her and the other sexually assaulted her
Fairness	Bastard they all seem to use mental illness as a defence for Terrorism this is inexcusable and the law needs to be changed so these Moslem Scum that Commit Terrorist Attacks Don't get away with their crimes. The Law is an Ass!
	Britain is a country where a rich black man can set up a university scholarship for black people ONLY & if you question if it's 'racist' YOU are 'Racist'. But if a white man set one up for whites only, there would be no doubt it's 'racist' & he would likely be prosecuted for race hate.
	There should be a movement to remove womens right to vote! They just cant think logically!

	No pension for traitors....just lethal injections!
Loyalty	Anyone surprised that another clueless May appointee hates the white working class while loving immigrants.
	Also as property tax may still be low there, is there a way around the niggertax and possibly a separate or new city council that serves white people? Meaning„all the property tax goes towards the white community
	Yeah fucking right Donald, you ran because you were bailed out by a high ranking (((Jew))) financially which made you in debt to the (((Jews))). You are clearly not interested in helping America and especially white Americans.
Authority	Britain is a country where a rich black man can set up a university scholarship for black people ONLY & if you question if it’s ‘racist’ YOU are ‘Racist’. But if a white man set one up for whites only, there would be no doubt it’s ‘racist’ & he would likely be prosecuted for race hate.
	Go along until you have taken all seats of power, vote each other into office, kick out the catlady lesbian and burn the BLM flag. Purge the entire thing. You now control the assets of the church, have a nice place for your homeschoolers to meet too. And you will get free media attention from the hate. If you’re not hated by the satanists, you’re not a Christian.
	F****ts defiling the graves of war dead. Welcome to Bosnia/Croatia? FFS. Morons
Purity	Amen! The Devil is real and He’ s a Democrat!

Never knew their Talmud states it is ok to rape 3 yr olds. This is sic & perverted.

Classifier fitting procedure and performance

Each fine-tuned model was trained trained for 2 epochs using weighted losses (per predicted binary class) in order to account for label imbalance by up-weighting models' attention to positive cases. In 10-fold cross validation, performance for hate-based rhetoric can be found in Kennedy et al. (2022), and prediction performance for moral vices can be found in Table S5. In order to contextualize the performance of these models in predicting labels for out-of-sample posts, two baselines are also included in Table S5: a Support Vector Machine (SVM; Cortes & Vapnik, 1995) with dictionary-based features derived from the Linguistic Inquiry and Word Count (LIWC; Pennebaker et al., 2015), and an SVM trained with Term Frequency-Inverse Document Frequency (TF-IDF), a strong baseline for text classification.

Robustness tests for filtering Gab users

Fitting models to annotated dataset

To accompany our main analysis of the dataset of predicted moral sentiment and hate-based rhetoric, here we report fully on a similar analysis using the annotated dataset (n = 27,655). Doing so allows us to verify that using predicted labels did not result in a notable difference in findings than if we were to use the annotated samples, which would indicate that the classifier we used for generating predictions introduced unwanted noise.

Table S7 presents results of two logistic regressions, for Human Degradation (HD) and Call for Violence (CV) labels, using the five moral vice labels as independent variables. The coefficients are not directly comparable to the mixed-effect models reported in the main text, however, the coefficients suggest a near-identical relationship between moral sentiment labels and the two hate-based rhetoric labels. In particular, Purity

Table S5

Mean and SE of F_1, recall, and precision for 10-fold cross-validation of moral vice labels (Care, Fairness, Loyalty, Authority, and Purity) and hate-based-rhetoric labels (Human Degradation, Calls for Violence).

	**Method**	**F_1**	**Precision**	**Recall**
Care	Random	11.4 (0.2)	6.4 (0.2)	51.1 (1.0)
	SVM_{LIWC}	35.9 (1.1)	30.1 (1.4)	45.2 (1.3)
	$SVM_{TF\text{-}IDF}$	41.1 (0.8)	42.3 (2.3)	41.2 (1.5)
	BERT	44.4 (0.5)	43.4 (2.0)	47.1 (2.0)
Fairness	Random	6.7 (0.3)	3.6 (0.1)	51.1 (1.2)
	SVM_{LIWC}	8.2 (0.9)	5.4 (0.6)	17.6 (2.0)
	$SVM_{TF\text{-}IDF}$	24.1 (0.8)	18.9 (0.6)	33.7 (1.4)
	BERT	27.0 (1.0)	27.5 (1.5)	27.7 (1.6)
Loyalty	Random	3.7 (0.2)	1.9 (0.1)	49.3 (2.3)
	SVM_{LIWC}	9.9 (0.7)	6.7 (0.6)	19.6 (1.1)
	$SVM_{TF\text{-}IDF}$	19.0 (2.4)	22.1 (2.0)	17.3 (2.7)
	BERT	22.2 (1.7)	24.1 (1.7)	22.2 (2.7)
Authority	Random	3.3 (0.2)	1.7 (0.1)	51.8 (3.5)
	SVM_{LIWC}	10.0 (0.6)	6.1 (0.3)	28.0 (1.8)
	$SVM_{TF\text{-}IDF}$	13.6 (1.0)	9.8 (0.8)	22.5 (1.4)
	BERT	10.8 (1.5)	13.6 (2.2)	10.9 (2.0)
Purity	Random	7.7 (0.4)	4.1 (0.2)	50.2 (1.3)
	SVM_{LIWC}	23.0 (1.4)	16.0 (0.9)	41.3 (3.1)
	$SVM_{TF\text{-}IDF}$	27.8 (2.4)	27.4 (1.7)	29.4 (3.5)
	BERT	30.2 (1.2)	33.7 (2.8)	28.8 (1.3)
HD	Random	14.3 (0.4)	8.3 (0.2)	49.1 (0.9)
	SVM_{LIWC}	34.8 (0.8)	26.5 (0.8)	51.2 (1.6)
	SVM_{TF-IDF}	44.4 (1.3)	40.2 (2.1)	51.2 (2.2)
	BERT	50.4 (1.0)	44.7 (2.0)	59.8 (2.8)
CV	Random	1.1 (0.2)	0.6 (0.1)	48.6 (5.1)
	SVM_{LIWC}	11.2 (1.8)	9.3 (2.7)	20.1 (2.3)
	SVM_{TF-IDF}	11.6 (2.7)	22.0 (6.4)	8.5 (1.9)
	BERT	23.4 (2.6)	21.2 (3.2)	31.9 (3.2)

(Degradation) has the strongest predictive relationship with the presence of HD, while Care (Harm) has the strongest predictive relationship with the presence of CV.

Table S6

Alternate results of Gab regression analysis after excluding users with more than 500 posts (results in main text excluded users with more than 5,000 posts). All fixed effects were significant ($p < 0.001$).

	Dependent variable:	
	Human Degradation	Call for Violence
Fixed Effects		
Intercept	−3.359 (0.006)	−7.026 (0.012)
Care	1.187 (0.005)	3.982 (0.012)
Fairness	0.884 (0.005)	−0.643 (0.020)
Loyalty	1.321 (0.013)	1.402 (0.027)
Authority	0.142 (0.013)	−0.490 (0.039)
Purity	2.649 (0.005)	0.328 (0.012)
Random Effects		
$\text{Intercept}_{\text{User}}$	1.584	1.060
$\text{Harm}_{\text{User}}$	0.162	0.417
$\text{Cheating}_{\text{User}}$	0.150	0.179
$\text{Betrayal}_{\text{User}}$	0.450	0.790
$\text{Subversion}_{\text{User}}$	0.321	0.595
$\text{Degradation}_{\text{User}}$	0.263	0.168
Note:	Estimates reported on the log-scale.	

Table S7

Results of two logistic regressions (with HD and CV as the respective dependent variables) using the annotated dataset (n = 27,655).

	Dependent variable:	
	Human Degradation	Call for Violence
Intercept	−2.883***	−6.841***
	(0.028)	(0.190)
Care (Harm)	1.460***	4.255***
	(0.065)	(0.212)
Fairness (Cheating)	0.777***	−0.493
	(0.097)	(0.518)
Loyalty (Betrayal)	1.250***	0.605*
	(0.111)	(0.366)
Authority (Subversion)	0.252*	−0.852
	(0.147)	(0.724)
Purity (Degradation)	2.684***	0.424*
	(0.067)	(0.254)
Note:		*p<0.1; **p<0.05; ***p<0.01

Appendix D

*

References

Bible, H. (1989). Authorized king james version. *London & New York, nd.*

Bojanowski, P., Grave, E., Joulin, A., & Mikolov, T. (2017). Enriching word vectors with subword information. *Transactions of the association for computational linguistics*, *5*, 135–146.

Cortes, C., & Vapnik, V. (1995). Support-vector networks. *Machine learning*, *20*(3), 273–297.

Kennedy, B., Atari, M., Davani, A. M., Yeh, L., Omrani, A., Kim, Y., Coombs Jr., K., Havaldar, S., Portillo-Wightman, G., Gonzalez, E., Hoover, J., Azatian, A., Cardenas, G., Hussain, A., Lara, A., Omary, A., Park, C., Wang, X., Wijaya, C., … Dehghani, M. (2022). The gab hate corpus: A collection of 27k posts annotated for hate speech. *56*, 79–108. https://doi.org/10.31234/osf.io/hqjxn

McInnes, L., Healy, J., & Melville, J. (2018). Umap: Uniform manifold approximation and projection for dimension reduction. *arXiv preprint arXiv:1802.03426*.

Pennebaker, J. W., Boyd, R. L., Jordan, K., & Blackburn, K. (2015). *The development and psychometric properties of liwc2015* (tech. rep.).

Řehřek, R., Sojka, P., et al. (2011). Gensim—statistical semantics in python. *Retrieved from genism.org*.
